# Adolescents infer latent motives to build generalizable models of others’ behavior

**DOI:** 10.1016/j.isci.2026.118039

**Published:** 2026-10-27

**Authors:** Jaime Vigil-Escalera, Jeroen van Baar, Lucas Molleman, Anna C.K. van Duijvenvoorde, Ili Ma

**Affiliations:** 1 Donders Institute for Brain, Cognition and Behaviour, Radboud University, Thomas van Aquinostraat 4, 6525GD Nijmegen, the Netherlands; 2 Department of Epidemiology, Boston University School of Public Health, 715 Albany St, Boston, MA, USA; 3 Department of Psychology, University of Amsterdam, Nieuwe Achtergracht 129-B, 1018 WT Amsterdam, the Netherlands; 4 Department of Developmental and Educational Psychology, Institute of Psychology, Leiden University, Wassenaarseweg 52, 2333AK Leiden, the Netherlands

**Keywords:** adolescence, decision-making, social decision making, reinforcement learning, computational modelling, social development

## Abstract

Understanding and predicting others’ behavior is a key developmental task in adolescence as social interactions become more complex. Because social situations rarely repeat exactly, accurate prediction requires generalization beyond past actions. We examined how adolescents (*n* = 127; ages 10–20) predicted others’ behavior across a series of economic games. Prediction accuracy improved with age. Computational modeling showed that this improvement was driven by an increasing tendency to infer unobservable motives underlying behavior (e.g., greed and risk aversion), with older adolescents more likely to infer multiple motives. In particular, inferring risk aversion became more common from early to mid-adolescence. These results suggest that adolescents increasingly build abstract, generalizable models of others’ behavior, supporting flexible social reasoning in novel contexts.

## Highlights

• Adolescents inferred latent, generalizable motives to predict others’ social decisions
• The types of motives inferred by adolescents matched those commonly observed in adults
• The number of distinct motives inferred by adolescents increased with age

## Graphical abstract

**Figure:**
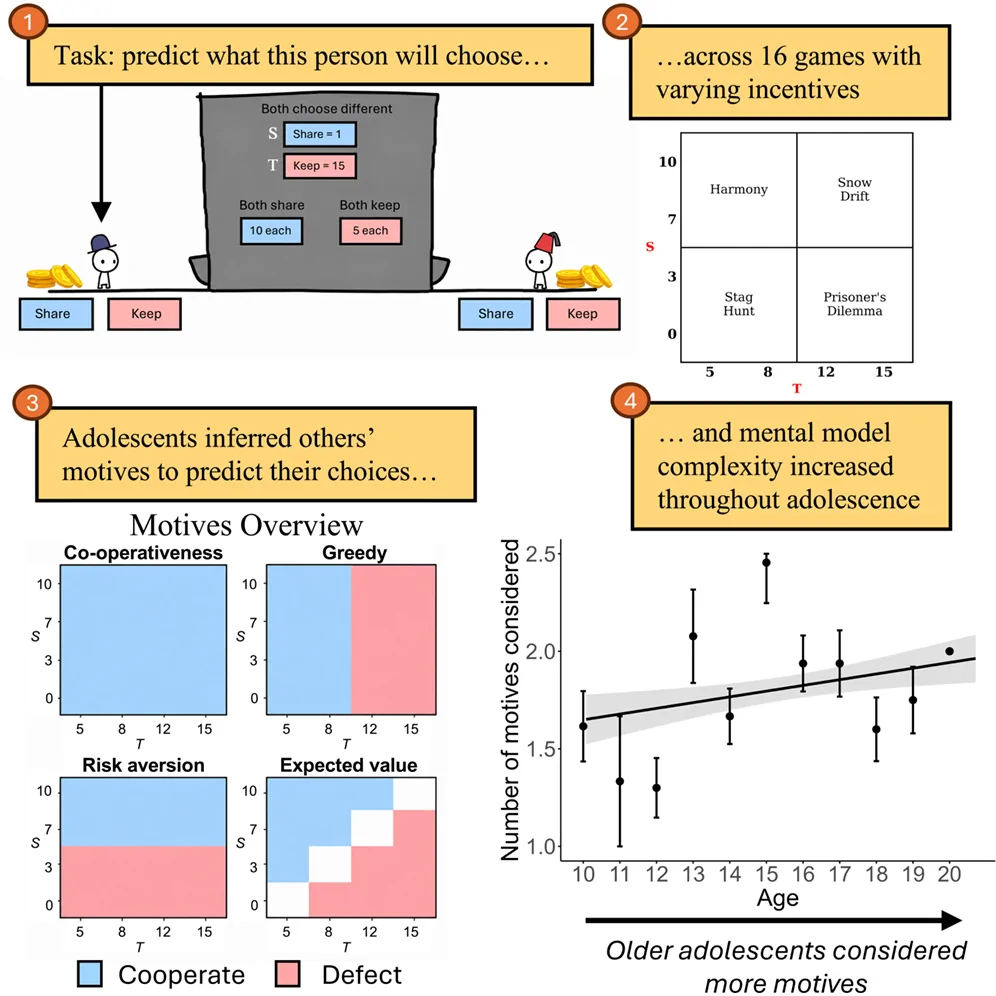

## Teaser

Natural sciences; Biological sciences; Neuroscience; Behavioral neuroscience; Cognitive neuroscience

## Introduction

Social interactions are rarely identical. The same person may behave differently depending on the context: a generally cooperative individual may refuse to help when the personal cost is high, while a reserved person may act boldly if their reputation is at stake. Successfully navigating such variation requires not only recognizing how someone typically behaves but also inferring the motives that drive their choices. Adolescence is a key period for refining these social inference skills, as peer relationships become more central and social environments become more complex,^1^^,^^2^^,^^3^ making learning about others more salient.^4^ Yet most developmental research has examined how adolescents detect others’ general behavioral tendencies (e.g., cooperativeness) rather than the underlying motives shaping others’ actions. Here, we define motives as stable preferences and personality traits that encompass both moral and non-moral concerns and form the building blocks of our mental models of others’ behavior.^5^ We use a structure-based reinforcement learning approach to examine how adolescents infer social motives, such as risk aversion, that enable accurate prediction of others’ behavior in new situations.

From a computational perspective, “motive inference” can be placed within a broader goal-directed learning framework. In many models of social learning, beliefs about others’ behavior are updated by directly associating the probability of a specific action with an individual (e.g., learning that a person is typically trustworthy) while keeping the context in which those decisions are made the same. Structure learning, by contrast, operates at a higher representational level: it identifies latent variables, such as motives, that consistently generate others’ behavior across contexts. Once inferred, these latent motives compress rich behavior into a compact set of latent variables and enable generalization beyond situations that have been experienced in the past. Motive inference thus complements traditional social learning by providing a compressed foundation for flexible, context-sensitive predictions.

Such context-sensitivity is a crucial element of positive social interactions. For example, a teenager may need to decide whether or not to confide in a friend. If their decision is solely based on how trustworthy that friend has been in the past, they might fail to anticipate that trustworthiness is not absolute. A friend who usually keeps secrets in private may gossip if they stand to gain social approval. Understanding such underlying motives that others may have, for example, whether they prioritize loyalty or personal gain, allows for adaptive social decision-making across different contexts (e.g., to avoid betrayal). Inferences of generalizable motives are particularly important during adolescence, as adolescents place greater importance on social status and popularity compared to children and adults.^6^

Despite its importance, most experimental work has examined how adolescents learn what decisions another person typically makes in repeated, identical settings.^7^ These studies show age-related changes in learning from social feedback, such as greater reliance on actively sampled information about a partner’s past behavior,^4^^,^^8^ increased avoidance of repeatedly untrustworthy partners,^9^^,^^10^^,^^11^ and better correction of inaccurate prior information.^12^ However, these studies tend to ignore that the context in which social decisions are made often varies in real-world interactions. Accurately predicting behavior in novel situations requires generalizing from past experiences by inferring underlying motives.^5^^,^^13^^,^^14^

In adults, van Baar et al.^5^ showed that people infer motives such as risk aversion and greed from others’ decisions in economic games. Crucially, adults use these inferences to predict others' behavior in previously unseen games. In their paradigm, participants observed another person’s decisions in games with systematically varied costs of cooperation and potential gains from defection. Rather than forming a fixed impression of trustworthiness, participants adapted their predictions based on the specific structure of each situation, which resulted in more accurate predictions. Computational modeling revealed that adults inferred motives such as greed and risk aversion to make generalizable predictions. This framework provides a powerful tool for examining how social predictions are formed during adolescence.

The motives that people infer likely align with well-established behavioral phenotypes in economic decision-making research.^5^^,^^15^^,^^16^ One such example is expected value (EV) maximization, a foundational principle in economic decision theory that has been shown to guide behavior across a wide range of social and non-social contexts. EV maximizers select actions by integrating probabilistic information about potential outcomes, such as the likelihood of cooperation, and choosing the option with the highest expected utility.^17^ Other common motives in social decision-making games include cooperativeness, where joint outcomes are prioritized even at personal cost^18^; risk aversion, the avoidance of uncertain outcomes when there is a potential large loss^19^; and greed, the pursuit of personal gain regardless of others’ outcomes.^20^ Importantly, these motives are not just descriptive labels but reflect decision rules applied across contexts. Computational modeling allows us to test if adolescents indeed infer such underlying motives to make social predictions as well as identify which motives they infer.

The set of inferred motives may expand during adolescence, reflecting greater experience with social interactions as well as their increasing complexity and salience.^1^^,^^4^^,^^21^ The ongoing development of cognitive empathy may further contribute to this development, as cognitive empathy reflects the ability to understand and reason about another person’s thoughts, intentions, and motivations, particularly when they differ from one’s own.^9^^,^^22^^,^^23^^,^^24^^,^^25^ One type of bias that may be overcome by the development of cognitive empathy is the “self-anchoring” strategy, where individuals project their own motives onto others. For example, risk-averse adults are more likely to assume that others are similarly risk-averse.^5^ Throughout adolescence, individuals may become better at considering multiple motives that differ from their own when predicting others’ behavior.^26^^,^^27^

Individual differences in adverse social experiences may meaningfully shape expectations about others’ motives. For instance, adolescents with elevated social anxiety or exposure to aggression may develop heightened sensitivity to social threat.^28^^,^^29^^,^^30^^,^^31^ Such experiences could bias the set of motives that an individual is likely to infer. For example, repeated exposure to untrustworthy or exploitative behavior might make adolescents more likely to expect others’ actions to be driven by greed or self-interest, even when alternative motives (e.g., risk aversion and fairness) could explain the behavior. This selective bias toward particular motives could hinder the formation of accurate, generalizable motivational representations. Consequently, such biased inferences may reinforce negative expectations and constrain flexibility in social learning.

In this preregistered study, we addressed four aims. First, we assessed whether adolescents infer latent motives that are known to drive people’s social decisions. To distinguish latent motive inference from learning in general, we tested whether adolescents are better at predicting behaviors that match naturally occurring human motives than those arising from artificial, non-human motives. Second, we examined if adolescents use the same generalization strategy as adults to make social predictions. This was tested by fitting computational models that each represented a different generalization strategy for making social predictions and comparing their fits. Third, we investigated whether the set of inferred motives varies with age. Fourth, we investigated whether individual differences in cognitive empathy, self-anchoring, social anxiety, and prior aggression predict variation in inferred motives. We expected motive inference to improve during adolescence and used computational models of motivation-based reinforcement learning to capture the inference process.

## Results

One hundred twenty-seven adolescents (age range = 10–20 years) performed the social prediction game (Figure 1A), in which they were asked to predict the choices of four distinct players across 16 trials of four distinct types of economic games (Figure 1B). During each trial, the player decides whether to cooperate or defect, following a strategy that results in a unique response pattern for each player across the four types of games (Figure 1C). The task is designed so that each player cooperates on 50% of trials, meaning that accuracy significantly above that threshold would indicate learning about players and generalizing this information across trials.

**Figure 1:**
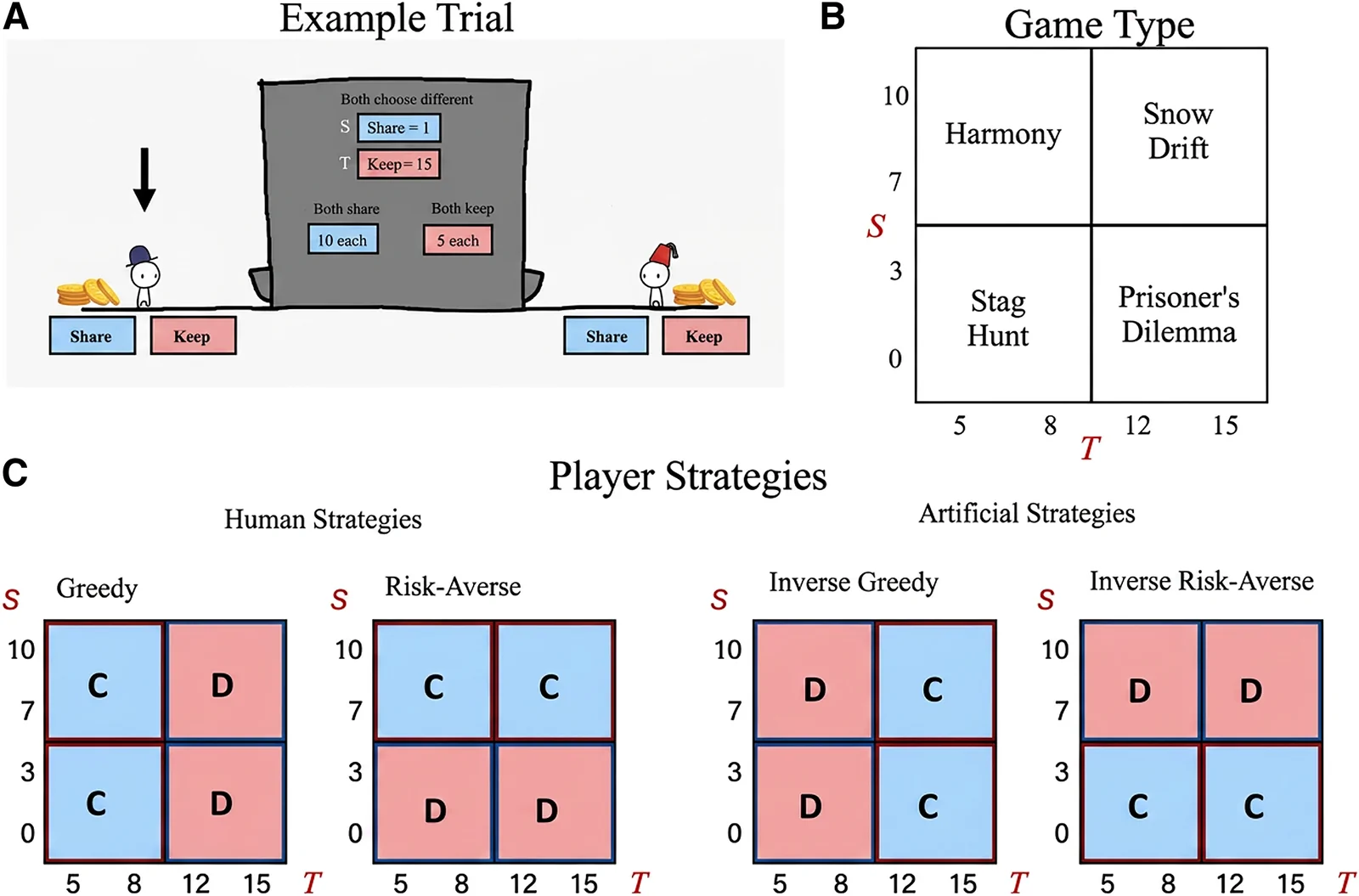
The social prediction game (A) An example trial from the social prediction task as presented to participants. In each trial, two players are shown on opposite sides of a machine, each with an endowment of five coins. The players must choose either to keep or share their coins by inserting them into the machine. The machine displays all possible outcomes based on both players’ choices. The two outcomes at the top of the machine, marked as S (sucker’s payoff) and T (temptation to defect), varied between trials. The letters S and T were not shown to participants but are shown for clarification purposes in this figure. Based on this information, participants predicted the choice of the leftmost player (player 1, marked by the arrow) and then indicated their confidence in their prediction on a scale from 0 to 100. Player 1 on the left remained the same for 16 trials, while player 2 on the right was unique on each trial. (B) The horizontal axis displays the values of T (temptation to defect) across the 16 games, while the vertical axis shows all possible values of S (sucker’s payoff). The division into four quadrants illustrates how the combination of S and T maps to the four types of economic games used in the social prediction task. This figure was adapted from Van Baar et al.^5^ (C) Each matrix represents the response pattern of one of the four players in the social prediction task, in which “C” denotes a cooperation response (i.e., sharing) and “D” represents defection (i.e., keeping). The horizontal axis displays all possible values of T (temptation to defect) across the 16 games, while the vertical axis shows all possible values of S (sucker’s payoff). This figure was adapted from one used by Van Baar et al.^5^

### Behavioral results

To test whether participants generalized information between trials, we first compared average task accuracy against the 50% chance level. Confirming our hypothesis, participants performed significantly above chance (M = 58.5%, SD = 22.3%, *t*(507) = 8.56, *p* < 0.001, d = 0.38; Figure 2A). Then, to test whether participants were leveraging their prior expectations to inform their predictions, we compared their prediction accuracy when predicting the choices of players following human motives and players following artificial motives. As expected, participants were more accurate when predicting behavior in human-motive conditions (M = 74.5%, SD = 15.4%) than in artificial-motive conditions (M = 42.4%, SD = 15.7%, mean difference = 32.1%, *t*(253) = −26.58, *p* < 0.001, d = −2.04; Figure 2B, and Table 1).

**Figure 2:**
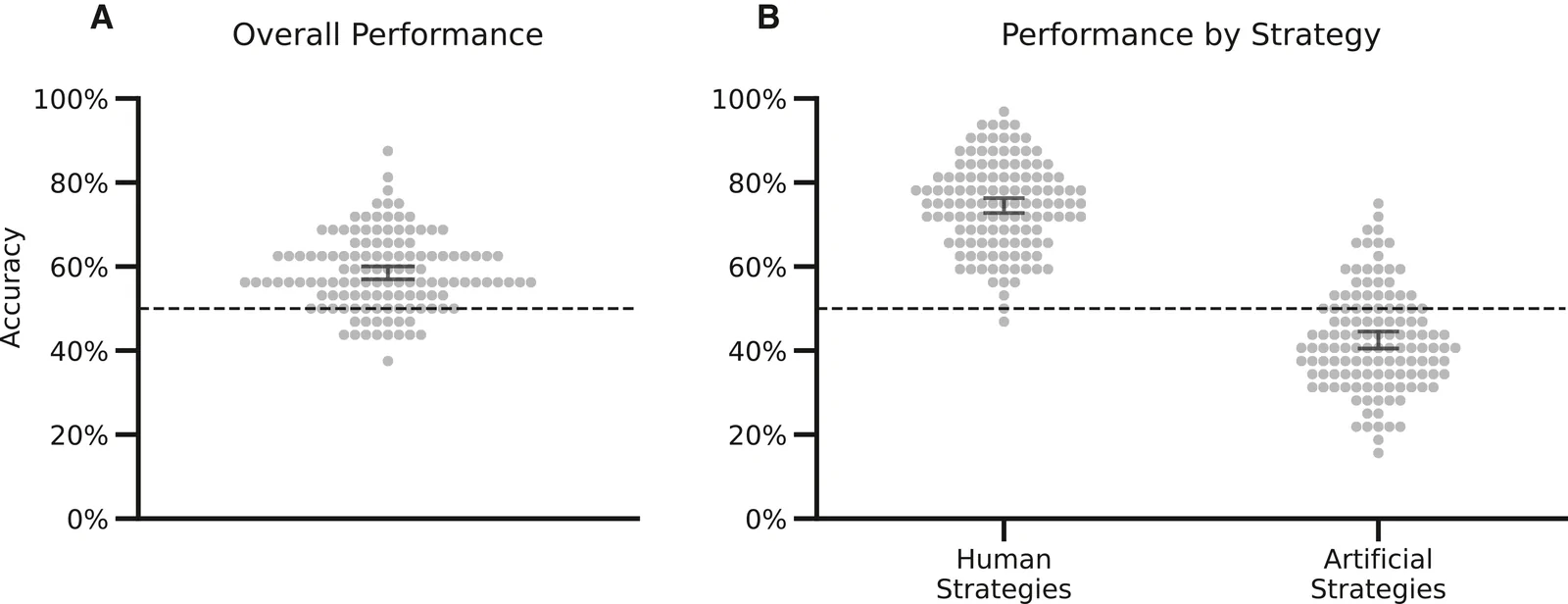
Prediction accuracy (A) Overall task performance: each dot represents an individual participant’s total accuracy across all four blocks, and the sample standard deviation is marked with a black error bar at the center of the distribution. The dotted line indicates the 50% threshold. Together, these elements show a total average performance significantly above 50%. (B) Comparison between accuracy with human motives and accuracy with artificial motives. Accuracy in both conditions significantly differ from 50%, and is significantly different between conditions.

**Table 1:** Mean accuracy per player type

	M	SD
Greed	73.7%	15.9%
Risk aversion	75.3%	15.0%
Inverse greed	41.6%	15.1%
Inverse risk aversion	43.4%	15.5%

Group level proportion of correct predictions for each of the four player types.

Next, we tested the pre-registered hypothesis that participants’ predictions would become more accurate over time (operationalized as trial number) and examined whether age played a role in this (Figure 3). As expected, we found that participants became more accurate over time (odds ratio [OR] = 1.17, 95% confidence interval [CI] [1.09–1.26], β = 0.157, *p* < 0.001). Furthermore, in line with our main pre-registered hypothesis, we found that older participants were significantly more accurate than younger participants (OR = 1.11, 95% CI [1.01–1.23], *β* = 0.108, *p* < 0.05). The interaction between age and time was not significant (OR = 1.03, 95% CI [0.96–1.10], *β* = 0.028, *p* = 0.43). Quadratic and cubic age effects were also tested, but neither significantly improved the model fit.

**Figure 3:**
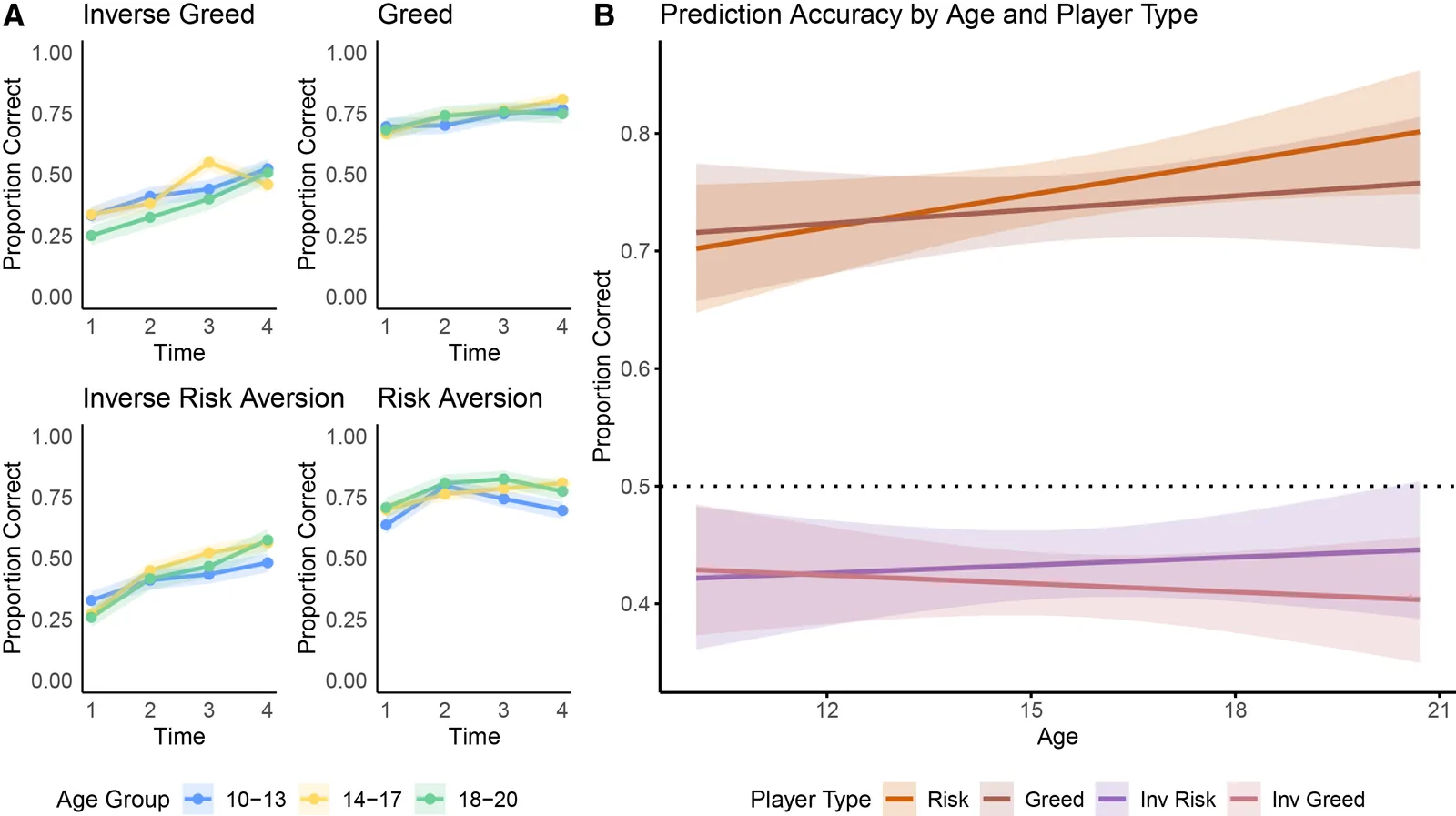
Learning across trials by player type Each plot shows the proportion of correct responses per trial for each of the four player types (i.e., greed, inverse greed, risk aversion, and inverse risk aversion). The lines represent the mean, and the shaded regions represent the standard error of the mean. (A) Proportion of correct responses as a function of time. Each unit on the horizontal axis represents four consecutive trials in which all game types were presented in random order. The corresponding plotted value shows the average proportion of correct responses across these trials. Age groups were used for visualization purposes only. (B) Proportion of correct predictions as a function of age for each player type. The dotted line indicates the chance level at the 50% threshold.

We then tested whether accuracy differed between the four different players presented in the task. As hypothesized, accuracy differed between the players (F(3,378) = 199.7, *p* < 0.001). To understand which player conditions were driving this effect, we conducted post hoc pairwise comparisons. We found that accuracy was significantly lower when predicting the choices of either inverse player type as compared to either human player type (inverse-greedy vs. greedy: β = −0.321, SE = 0.0185, *t*(378) = −17.35, *p* < 0.0001; inverse-greedy vs. risk: β = −0.337, SE = 0.0185, *t*(378) = −18.20, *p* < 0.0001; inverse-risk vs. greedy: β = −0.303, SE = 0.0185, *t*(378) = −16.37, *p* < 0.0001; inverse-risk vs. risk: β = −0.319, SE = 0.0185, *t*(378) = −17.22, *p* < 0.0001). Accuracy did not differ between the two human motives (greedy vs. risk: β = −0.0157, SE = 0.0185, *df* = 378, *t* = −0.850, *p* = 1.000) or between the two inverse player types (β = −0.0182, SE = 0.0185, *df* = 378, *t* = −0.983, *p* = 1.000). Additionally, we found highly significant main effects of game type (*F*(3, 1890) = 17.38, *p* < 0.001), and player type (*F*(3, 1890) = 274.11, *p* < 0.001), as well as the interaction between game type and player type (*F*(9, 1890) = 64.36, *p* < 0.001) on overall prediction accuracy (Figure S1).

Participants' self-reports of each player’s motive confirmed that participants interpreted the greedy player as personal gain maximizing, the risk-averse player as self-interested but cautious, and the players withsupp inverse motives as difficult to interpret (see manipulation check: self-reported inferred motives). This supports our assumption that participants’ perception of the players was consistent with their pre-programmed motives.

### Computational model comparisons

Model comparisons (Figure 4) showed that the full motivation-based model (Bayesian information criterion [BIC]: M = 35.3, SD = 31.3), which includes all four motives tested, outperformed both the no-structure model (BIC: M = 86.0, SD = 31.6; full motive vs. no structure: wins = 122, losses = 5, 95% CI [bootstrap] = [46.69, 54.71]) and the game type-based model (BIC: M = 44.4, SD = 29.7; full motive vs. game types: wins = 115, losses = 12, 95% CI [bootstrap] = [7.50, 10.84]). Together, these findings show that the full motivation-based model outperformed both the no structure and game type-based models at the individual level (i.e., won the pairwise comparisons for nearly all participants) and at the group level (i.e., it had a lower mean BIC, with a CI difference that excluded zero, indicating a reliable difference in model fit). Model fit was further improved by the motivation subset-based model (BIC: M = 23.9, SD = 31.3), which includes only a subject-unique subset of the four motives tested, and which in turn outperformed the full motivation-based model (full motive vs. subset motive: wins = 0, losses = 126, 95% CI [bootstrap] = [10.14, 12.78]). This result was confirmed by a random-effects Bayesian model selection (RFX-BMS) analysis,^32^ which strongly favored the motivation subset-based model (*r* = 0.99, protected exceedance probability (PEP) ≈ 1.00, Bayesian omnibus risk (BOR) <0.001).

**Figure 4:**
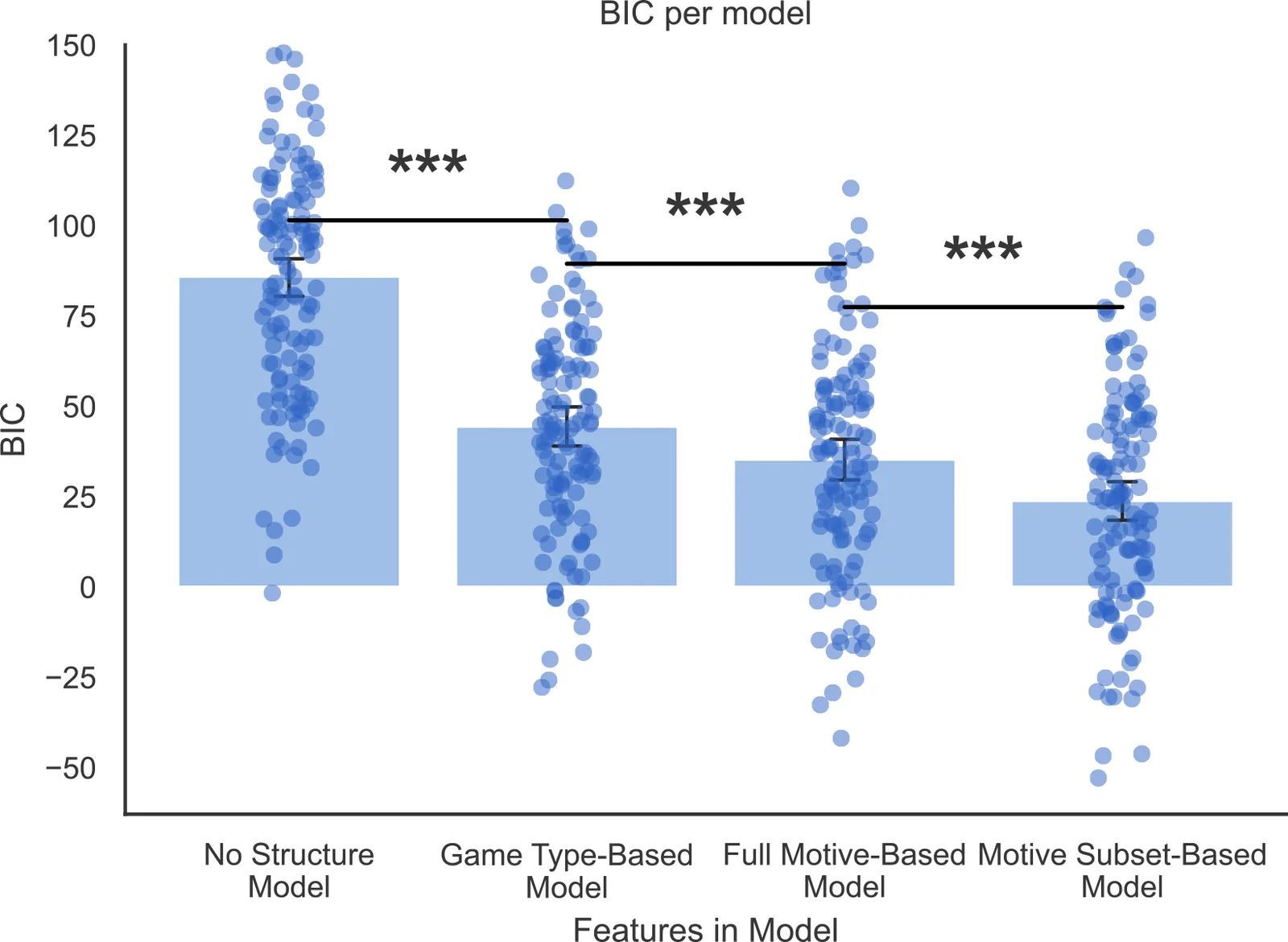
BIC Comparison for each model Blue dots indicate BIC values at the individual level, while bars represent the mean BIC per model; error bars indicate the standard deviation. Lower BIC values correspond to better model fit. For each pairwise comparison, the per-participant ΔBIC was computed and its reliability assessed using a percentile bootstrap (10,000 resamples); statistical significance is indicated as follows: ∗95% bootstrap CI excludes zero, ∗∗99% bootstrap CI excludes zero, ∗∗∗99.9% bootstrap CI excludes zero. The winning model was the subset variant of the motivation-based model, which considers a subject-specific subset of the four motivations.

We repeated the model comparisons using the sum of squared errors (SSE) instead of BIC to check if the subset-based model fitted better than the full motivation-based model due to the penalty that BIC assigns to the number of free parameters. Reassuringly, the subset-based model (SSE: M = 4.80, SD = 2.47) showed smaller SSEs than the full motivation-based model (SSE: M = 4.95, SD = 2.47; full vs. subset: wins = 59, losses = 67, 95% CI [bootstrap] = [0.06, 0.25]), providing further evidence that participants consider some, but not all, of the four motives accounted for in our analysis to inform their predictions.

### Age differences in inferred motivations

Having fitted the computational models to each participant’s data, we could investigate whether and how the motivations inferred differed between subjects. We first evaluated how many motivations were included in each subject’s best-fitting motivation subset. Considering two motivations was the most common across our sample (Figure 5), which replicates the findings of the prior study in adults (van Baar et al., 2021). As expected, the age-related results show that the likelihood of considering more than one motive increased with age (i.e., with age there was a decreasing likelihood of considering only one motive, β = −0.41, SE = 0.19, z = −2.11, *p* = 0.035). Age was not significantly associated with the likelihood of considering more than two (β = 0.01, SE = 0.25, z = 0.05, *p* = 0.96) or three (β = 1.28, SE = 1.16, z = 1.10, *p* = 0.27) motives (Table S2). These findings suggest that the mental model used by participants to inform their predictions increased in complexity (i.e., the number of motives considered) as a function of age. Quadratic and cubic age effects did not significantly improve the model fit.

**Figure 5:**
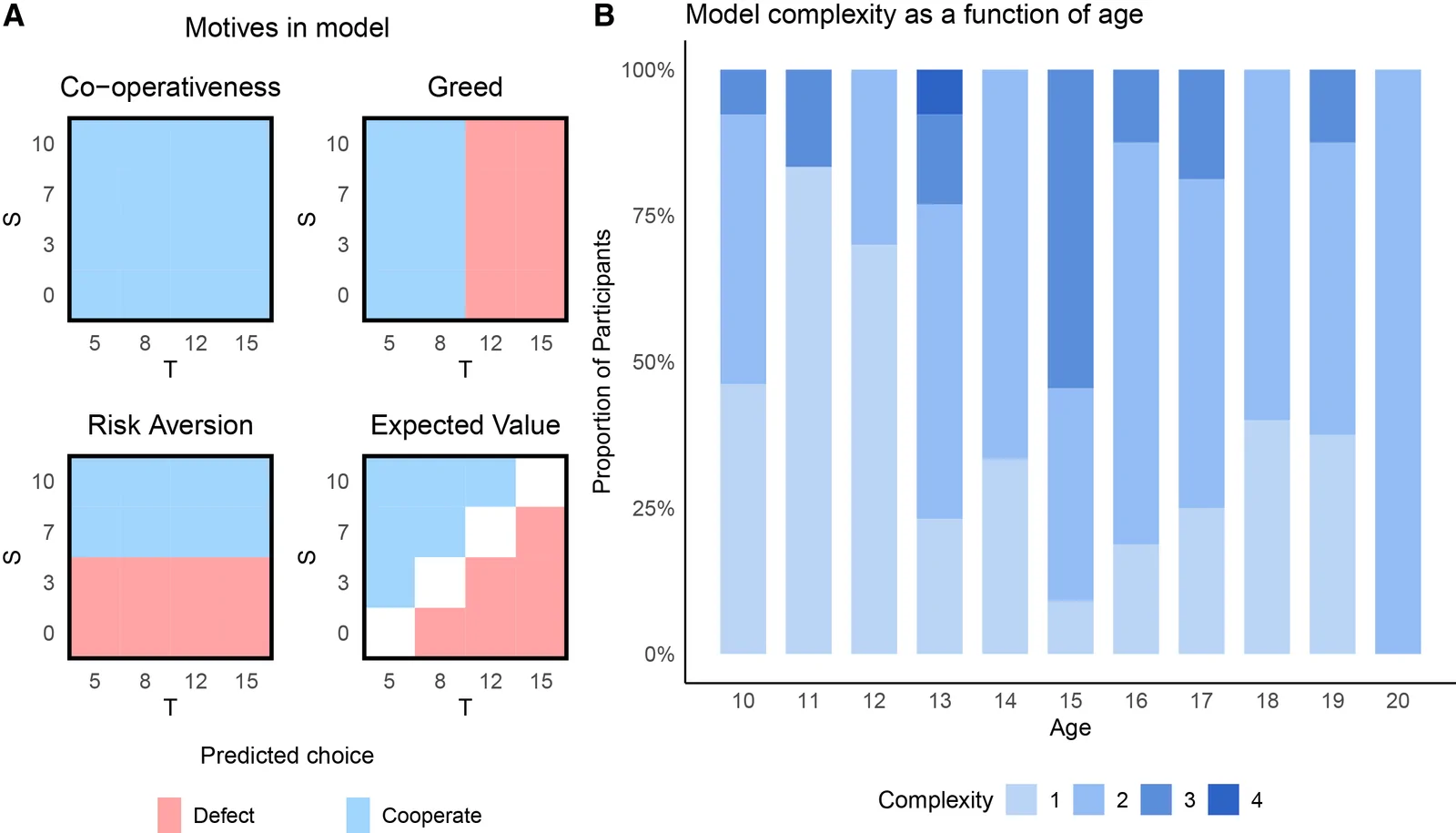
Changes in mental model complexity as a function of age (A) The predicted behavioral pattern from each of the four motives included in the winning model across the game space (S and T). (B) Complexity refers to the number of motivations included in each subject’s best-fitting motivation subset. Age was used as a continuous variable in all analyses and was only rounded and treated as a categorical variable for visualization purposes.

Next, we explored if a specific motive may be driving the age-related increase in model complexity. For this purpose, we evaluated which specific motives participants were more likely to infer according to the motivation subset-based model. The motivation most often inferred by participants was EV, which was included in the model for 64% of participants. This was followed by risk aversion, inferred by 49% of participants; greed, inferred by 45%; and cooperativeness, inferred by 24% of participants. This pattern differs from what was observed by Van Baar et al. (2021), who found that 87% of adults in their sample considered greed, 51% considered cooperativeness, 31% considered EV maximization, and only 21% considered risk aversion. Additionally, we found that the probability of inferring certain motivations changed as a function of age (Figure 6). Specifically, the probability of inferring risk aversion as a motivation underlying player 1’s decision increased with age, particularly from early to mid-adolescence (age = linear, estimate = 1.63, *p* = 0.028, OR = 5.10, 95% CI [1.26, 23.75]; age = quadratic, estimate = −0.047, *p* = 0.045, OR = 0.95, 95% CI [0.91, 0.998]), consistent with our hypothesis. However, unexpectedly, the probabilities of inferring the other motivations were not associated with age (greed: age = linear, estimate = 0.024, *p* = 0.688, OR = 1.02, 95% CI [0.91, 1.15]; EV: age = linear, estimate = 0.001, *p* = 0.983, OR = 1.00, 95% CI [0.89, 1.13]; cooperativeness: age = linear, estimate = −0.092, *p* = 0.197, OR = 0.91, 95% CI [0.79, 1.05]). Together, the results show that our main hypothesis was partially confirmed; the ability to infer the motives of others improved with age and this was particularly driven by an improvement in inferring risk aversion from early to mid-adolescence.

**Figure 6:**
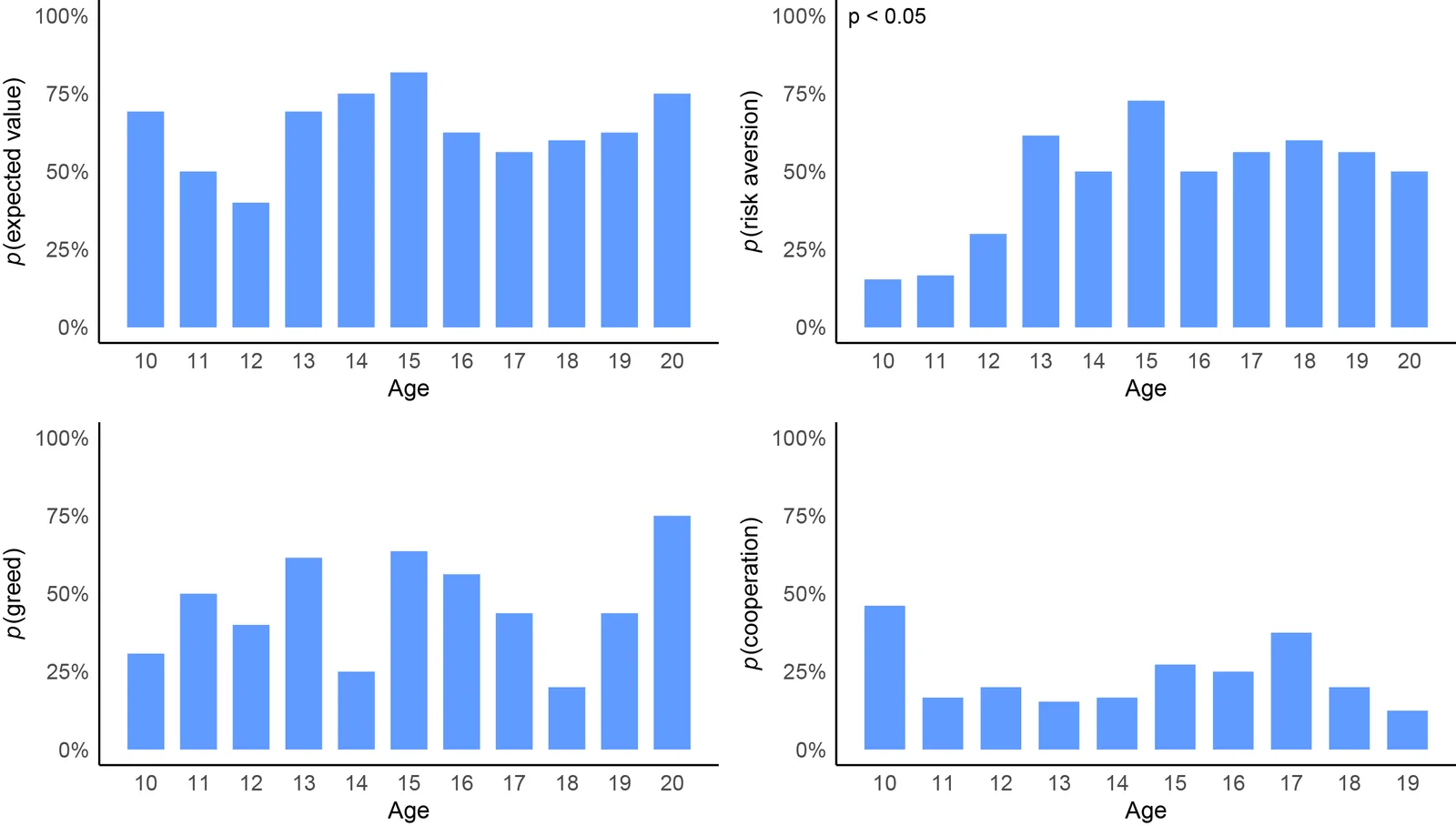
Probability of considering each motivation plotted as a function of age The bars show the proportion of individuals who, according to the motivation subset-based model, considered a specific motivation (i.e., expected value, risk aversion, greed, and cooperativeness) as a function of age. Age groups were used for visualization purposes only, as age was treated as a continuous variable in all analyses.

### Self anchoring results

Among the 127 participants, 20 followed the risk-averse motive, 16 the EV motive, 16 the greed motive, and 12 the cooperativeness motive. The remaining 63 participants did not match any specific motive pattern. The frequency of each motive changed as a function of age, which is summarized in Table 2. The full model results can be found in Figure S2 and Table S1.

**Table 2:** Frequency of own motive as a function of age

Age group	Coop	Greed	EV	Risk	None
10–13	5	3	7	3	24
14–17	3	11	7	12	22
18–20	4	2	2	5	17
Total	12	16	16	20	63

Age was treated as a continuous variable in all analyses.

We then tested whether self-anchoring led to increased task performance, such that participants were more accurate when predicting the choices of players who had the same motive as their own. Contrary to our pre-registered expectations, we did not find a significant effect of self-anchoring on prediction accuracy for risk averse players *b* = −5.12, *t*(123) = −1.18, *p* = 0.238) or greedy players (*b* = 9.8, *t*(123) = 1.85, *p* = 0.065). The interaction between self-anchoring and age was also not significant (for risk aversion, *b* = 0.36, *t*(123) = 1.37, *p* = 0.174; for greed, *b* = −0.62, *t*(123) = −1.88, *p* = 0.063).

Finally, the multinomial logistic regression analysis revealed significant linear and quadratic age effects on all the motives tested (Table S1). Suggesting that participants’ own preferred motives during the economic games varied with age (Figure S2). However, the scarcity of specific player motives and the limited number of trials used to determine player motives likely introduced noise. Therefore, more research is needed to assess age-related changes in own motives during economic games.

### Individual differences results

Contrary to our expectations, we found no significant relationships between mental model complexity and environmental harshness (pHolm = 1.0, OR = 0.97, 95% CI [0.91, 1.03]), social anxiety (pHolm = 0.5, OR = 0.97, 95% CI [0.95, 1.00]), or cognitive empathy (pHolm = 1.0, OR = 1.02, 95% CI [0.94, 1.11]). We also found no significant relationship between social anxiety and being risk-averse (pHolm = 0.4, OR = 0.96, 95% CI [0.92, 0.99]). Environmental harshness and cognitive empathy were similarly not significantly related to preferring the risk-averse motive (pHolm = 1.0, OR = 1.07, 95% CI [0.95, 1.21]). Individual differences are reported in Table 3.

**Table 3:** Individual differences

	M (range)	SD
Social anxiety	48.79 (22–110)	15.45
Environmental harshness	28.98 (15–89)	6.34
Cognitive empathy	24.03 (7–35)	4.88

Social anxiety refers to total score in the social anxiety scale for adolescents; environmental harshness is the combined score of the neighborhood violence scale and the parental aggression scale. Cognitive empathy refers to the perspective taking subscale of the interpersonal reactivity index.

## Discussion

Accurately predicting the behavior of others is a central part of social-cognitive development, particularly during adolescence, a period marked by heightened social salience and increasingly complex interpersonal goals.^1^^,^^4^^,^^33^ These changes increase the need to learn about generalizable aspects of others' behavior to accurately predict their behavior in novel settings. In this study, we show that adolescents can infer latent, generalizable motives that underlie people’s social decisions across contexts. These motives were not directly observable or communicated. Instead, they had to be inferred from behavior across multiple one-shot interactions that were observed from a third-party perspective. Moreover, the complexity of adolescents’ mental models about others’ motives increased with age. The findings extend prior developmental studies that position adolescence as a sensitive period for social learning^4^^,^^34^^,^^35^^,^^36^^,^^37^ by showing that adolescents build higher-order representations of “why” others act as they do. In this sense, our findings are consistent with the motive inference framework proposed in computational social learning.^5^

The conclusion that adolescents relied on latent motive inference is supported by several findings in our study. Adolescents were more accurate at predicting behavior when it was generated by common human motives compared with when it followed artificial, non-human motives. This suggests that their social predictions draw on a structured hypothesis space tuned to behavioral regularities that are likely to occur in real-world interactions. Model comparisons further revealed that adolescents’ predictions were best explained by latent motive inference of a subset of motives, similar as previously reported in adults.^5^ Models that did not include any structure, or in which the structure was determined by game-type did not account for adolescents’ predictions as well. Moreover, adolescents’ self-reports converged with our interpretation of the motives, as participants described player motives in ways that were consistent with greed and risk aversion. The generalizable structure that adolescents inferred to make predictions (i.e., greed and risk aversion) matched the inferred motives that are commonly observed in adult social decision-making^15^^,^^16^^,^^38^ and these representations supported cross-context generalization. This suggests that even in early adolescence, people are sensitive to deeper regularities in others’ behavior.

Importantly, our results also highlight age differences in motive inference. With age, adolescents were more likely to consider more than one motive. Inferences of risk aversion improved from early to mid-adolescence (10–15 years). This finding is consistent with studies showing a developmental refinement in the representational complexity of social inference in childhood^39^^,^^40^^,^^41^^,^^42^^,^^43^ and evidence that this refinement further develops in adolescence.^4^^,^^44^^,^^45^^,^^46^ Computational accounts show that humans use inverse planning from infancy (i.e., reasoning backward from observed actions to underlying goals and preferences).^40^^,^^47^^,^^48^^,^^49^ However, this literature has largely focused on simple inferences from physical movement, such as reaching or object-directed action.^50^^,^^51^^,^^52^ Our findings further extend these insights by demonstrating that adolescents show related inferential capacities when observing strategic social interactions. This supports the idea that social learning continues to undergo qualitative elaboration during adolescence.

Interestingly, the motives that adolescents inferred did not fully mirror those previously observed in adults. In our study, EV maximization was among the most prevalent inferred motives. Yet the adult participants in van Baar et al.^5^ most commonly inferred greed. This difference may point to an age-related shift in the baseline from which individuals evaluate the social decisions of others. In the social prediction game, the EV maximization strategy is to cooperate unless the temptation to defect is five coins higher than the sucker’s payoff, with five coins representing the outcome if both players keep. By contrast, the greedy strategy is to cooperate unless the temptation to defect exceeds ten coins, which is the outcome if both share. This distinction raises the possibility that adolescents expect others to make social decisions by comparing the temptation to defect to the outcome of mutual mistrust, whereas adults may rely on a more optimistic baseline anchored to the outcome of mutual cooperation. An adapted task design could test this directly, and in doing so, reveal further developmental changes in learning about others.

Self-anchoring (i.e., an egocentric bias in which one’s own motivations are projected onto others) was not a likely explanation for the more prevalent inference of EV maximization found in our study because it was not the most common motive in adolescents' own choices. In fact, contrary to expectations, we found limited evidence for self-anchoring. While null findings should be interpreted with caution as they may depend on task demands or perceived similarity between the participant and the observed agents, the finding may be theoretically informative. Self-anchoring is commonly observed in adult decision-making^53^ but adolescence may be a period in which people increasingly differentiate between their own perspective and that of others. Relying less on self-simulation may be adaptive, as it allows adolescents to readily learn that others think, value, and act differently from themselves.^4^ Future work is needed to test when adolescents rely on self-based simulation, other-oriented inference, or norm-based expectations when predicting others’ decisions.

Contrary to expectations, we also found no evidence that prior childhood exposure to aggression, cognitive empathy, or social anxiety predicted motive inference in this task. The social prediction task required participants to observe others from a third-party perspective, without being personally affected by the decisions. It is possible that the expected relationships emerge mostly in self-relevant contexts that engage affective processes.^54^ For example, adverse childhood experiences may influence learning strategies or bias the prior distribution over motives when participants’ own outcomes are at stake. Developing adequate motive-inference skills can be particularly relevant for youth who have been exposed to aggression, as these skills may support self-protective vigilance.^55^^,^^56^ Nevertheless, third-party observation is itself an ecologically valid and important mode of social learning and social competence. Much of what adolescents learn about social norms, reputations, and the character of other people occurs by observing interactions they are not directly part of, such as observing peer conflicts or following social dynamics within a group.^57^^,^^58^^,^^59^

Our study benefited from several strengths. The within-subjects manipulation of human and artificial motives allowed us to test whether adolescents were specifically sensitive to inferring human-like motivational structures from observed social decisions. Moreover, the randomization of trial and block order reduced potential order effects, and the use of computational modeling allowed us to distinguish latent motive inference from alternative models that produce similar levels of accuracy but imply different cognitive mechanisms. While online data collection may have affected participant engagement due to a lack of control over the online environment, we do not expect substantial qualitative differences due to the setting as the accuracy scores for the human motives were well above chance. Moreover, prior research established a high similarity between online and in-lab study results with adolescent participants,^60^ suggesting that our main conclusions are unlikely to be attributed to the test setting.

Together, our findings situate motive inference as a higher-level component of adolescent social learning. Adolescents inferred latent motivational structures that supported flexible predictions across varying contexts. The complexity of these representations increased with age, suggesting further elaboration of the cognitive models that adolescents use to understand the behavior of other people. This extends developmental research on adolescent social learning beyond a focus on context-independent action-outcome associations. Future work is needed to examine how these processes develop longitudinally and how they operate in less constrained environments where there is no full prior knowledge about the action and outcome space.^13^ Understanding these mechanisms will be essential for refining theoretical models of adolescent social development and potentially for informing interventions aimed at fostering social competence during this sensitive period for social development.

### Limitations of this study

The following limitations give rise to opportunities for future studies. First, data collection started during the later part of the COVID-19 pandemic, and it is therefore possible that social development or task performance could have been affected by experiences of social isolation.^61^^,^^62^^,^^63^ However, if social experience strengthens priors over human behavior, the current effects may underestimate adolescents’ motive-inference abilities under more typical social conditions. Future studies using quantitative measures of social interactions could provide further insights into this relationship. Second, due to a lack of power, we did not examine potential gender differences in prediction accuracy or model complexity. Future studies with large, gender-balanced samples are needed to examine whether developmental trajectories of social prediction differ by sex or gender. Third, our task requires learning about others’ decisions in a risk-taking context, i.e., a context where people may prefer a sure outcome over an uncertain outcome depending on the payoffs. It may be fruitful for future research to explore learning in other contexts such as those in which loss aversion may drive cooperative decision-making.^64^ Fourth, the cross-sectional design limits conclusions regarding within-person developmental change. Longitudinal studies with large sample sizes are required to test if adolescents become increasingly able to infer multiple motives over time and how such changes relate to real-world social experience, individual differences, and broader social competence.

A further question for future research is how motive inference in the social prediction task relates to real-world social competence. In the study by van Baar et al.,^5^ the adult participants played an inspection game after the social prediction task, in which they could pay to check whether an employee was shirking and withhold wages if so. Participants inspected employees they previously inferred to be greedy more often than those inferred to be risk averse. This suggests that people selectively generalize inferred motives to new situations, informing trustworthiness judgments. This insight may guide future studies testing how adolescents’ latent motive learning is associated with trustworthiness judgments in new contexts and real-life settings. Such studies could vary the social context to more closely approximate real-life social contexts.^8^^,^^65^^,^^66^

## Resource availability

### Lead contact

• For further information about the present study, please contact the lead contact, Dr. Ili Ma (i.ma@fsw.leidenuniv.nl).

### Materials availability

• This study did not generate new unique reagents. Task scripts and materials used generated in this study are available from the lead contact upon request.

### Data and code availability

• Raw and analyzed data have been deposited at OSF and are publicly available as of the date of publication. Accession numbers are listed in the key resources table.
• The code used for all analyses has been deposited at OSF and is publicly available as of the date of publication. Accession numbers are listed in the key resources table.
• This paper does not report additional items beyond the data and code listed above. Any additional information required to reanalyze the data reported in this paper is available from the lead contact upon request.

## Acknowledgments

We thank all our participants and their parents for their contributions. Many thanks to Daniël de Beer and all the BA and MA students who recruited the participants. We also thank Nicky Case for the game images as well as Willem Frankenhuis and Anne Miers for helpful discussions. L.M. is supported by the 10.13039/501100004359Swedish Research Council (grant no. 2024-01197). A.C.K.v.D. is supported by the 10.13039/501100003246NWO Gravitation program funded by the Dutch Ministry of Education, Culture and Science of the government of the Netherlands, grant no. 024.005.011. I.M. is supported by the 10.13039/100017325European Research Council (ERC StG 101164150-DISINFORMED TEENS).

## Author contributions

J.V.-E., formal data analysis, investigation, methodology, project administration, software, visualization, validation, and writing – original draft; J.v.B., conceptualization, investigation, methodology, software, supervision, validation, and writing – review and editing; L.M., methodology, validation, supervision, and writing – review and editing; A.C.K.v.D., methodology and writing – review and editing; I.M., conceptualization, investigation, methodology, resources, software, supervision, writing – original draft, and review and editing.

## Declaration of interests

The authors declare no conflict of interest.

## Declaration of generative AI and AI-assisted technologies in the manuscript preparation process

To summarize the participants’ open-ended answers to questions regarding their subjective interpretations of the motives, the authors used ChatGPT. For replicability and transparency, the procedure is fully described in the STAR Methods. The authors reviewed its output and take full responsibility for the content of the publication. None of the images used in the study or in the manuscript were produced by generative AI.

## STAR★Methods

### Key resources table

**Table:** 

REAGENT or RESOURCE	SOURCE	IDENTIFIER
**Deposited data**		

Raw and analyzed data	This paper	https://osf.io/p5h4j/

**Software and algorithms**		

MATLAB (R2022b)	MathWorks; https://www.mathworks.com/products/matlab.html	RRID:SCR_001622
Python	Python Software Foundation; https://www.python.org/	RRID:SCR_008394
R	The R Foundation; https://www.r-project.org/	RRID:SCR_001905
VBA toolbox	MBB-team; https://mbb-team.github.io/VBA-toolbox/download/	https://doi.org/10.1371/journal.pcbi.1003441 (no RRID registered)

### Method details

#### Participants

A total of *N* = 127 Dutch adolescents (age range = 10-20 years, M = 15.5, SD = 2.97, *n* = 55 men and *n* = 68 women, and four undisclosed) participated in our online study. Participants were recruited through schools and students’ social circles between 2021 and 2024. The study was approved by the Leiden University ethics review board (2021-01-11-I. Ma-V3-2759). Written informed consent was obtained from participants prior to participation. For minors, legal guardians also provided informed consent.

#### Pre-registration

The study was pre-registered prior to data collection ((https://osf.io/p5h4j/). Originally, the Pubertal Development Scale^67^ was included in the preregistration. However, our pilot showed that youth were uncomfortable with filling out these questions in our online survey as the scale asks about physical appearance as a proxy for pubertal stage. This scale was removed prior to data collection because of this reason and because pubertal stage was not a main focus of the study.

#### Study procedures

During the study, participants first viewed an audiovisual explanation of the Social Prediction Task. They then answered comprehension questions and played one trial of each game type present in the Social Prediction Task (prisoner’s dilemma, stag hunt, snow drift, and harmony game; see below). Participants were instructed that they would receive payment of 6 euros, plus up to an additional 4 euros based on their accuracy on randomly selected trials in the social prediction task. This was done to motivate their performance. In reality, all participants were paid 10 euros for their participation to avoid minors being disadvantaged by accuracy-based payment. After completing the social prediction task, participants filled out four questionnaires and were debriefed about the study’s objectives.

#### Social prediction task

In the Social Prediction Task,^5^ participants were asked to predict the decisions of others in a series of behavioral economic games, where players could either cooperate or defect, and participants had to predict what a given player would choose to do. In each trial, participants were shown an illustration of a machine with two cartoon characters, one on each side, representing other players (Figure 1A). The players were distinguishable by their unique hats. Next to each player were coins, representing monetary units, which they could either “share” with the other player (i.e., cooperate) or “keep” (i.e., defect). The goal for participants was to predict whether the player on the left side of the machine (player 1) would share or keep their coins (i.e., cooperate or defect), given the possible outcomes for both players. The machine displayed the potential payoffs, i.e., the monetary outcomes if both players cooperated, if both defected, and if they made different choices. This payoff matrix was presented in this way to make the information more accessible and the overall game structure and transitions between them more transparent and easier to grasp. On each trial participants first indicated their prediction (keep or share) and rated their confidence in their prediction on a scale from 0 to 100, then they saw player one’s choice and moved on to the next trial.

The outcomes were kept constant when both players shared (always 10 coins each) and when both chose to keep (always 5 coins each). The outcomes when each player made a different choice did vary across trials. On some trials the potential outcome for the player who shared was lower than the initial 5 coins, for example, in Prisoner’s Dilemma games. This lower payoff for the player who shared is sometimes referred to as the “sucker’s payoff” (S). In these trials, sharing reflects a risky choice. Similarly, the potential outcome for the player who kept was on some trials higher than the initial best outcome of 10 coins and is therefore sometimes referred to as the “temptation to defect” (T). Each trial had a unique combination of S and T, resulting in 16 unique trials for each player (16 x 4 trials in total for the entire task); the order was randomized between subjects to avoid order effects. Changing the values of S and T changes the incentives to cooperate. The combinations cover a range of strategically different situations that can be grouped into four game types that correspond with situations that are characteristic of human interactions.^13^ The first game type is the Prisoner’s Dilemma, which presents a situation where cooperation is risky due to a high temptation to defect (T) and a low payoff for the cooperator in an unequal interaction (S). The second game type is the Harmony Game, where cooperation is optimal for both players. The third game type is the Snow Drift, where unilateral defection results in the highest payoff, but mutual defection leads to a worse outcome. Finally, the fourth game type is the Stag Hunt, where mutual cooperation yields the highest payoff, while unilateral cooperation results in the lowest payout (Figure 1B). There were four payoff settings for each game type, leading to 16 trials in total per player.

After predicting 16 consecutive choices for one player, participants moved on to the next block, where they again predicted 16 choices of a new player, continuing until they had predicted the behavior of all four distinct players. Both the trial and block order were randomized. Each of the four players was programmed to respond differently to each game, according to a player-specific motivation that was sensitive to different combinations of S and T and was unknown to the participants (Figure 1C). The motivations of two players were based on behavior commonly found in humans in these types of games.^16^ The first human motivation used was one that was driven by personal gain, here called “greedy” which involved choosing the option with the highest possible payoff. The second motivation was “risk aversion”, in which players chose the option with the lowest risk of exploitation, defecting when the cost of unilateral cooperation became too high (e.g., when S = 0). Since each player cooperated in 50% of the trials, their choice was determined by the underlying motive, and trial order within the player type was randomized. As shown in Figure 1C, cooperative decisions in the range S<5 and T<10 and defection decisions in the range S>5 and T>10 dissociated the greedy and risk-averse players. The only way to perform better than chance on this task is to infer the underlying motive and use this to generalize predictions to new trials.

If adolescents indeed use naturally occurring latent motives to predict the decisions of others, then behaviors that match those motives should be easier to predict than behaviors that arise from artificial, non-human motivations. To test if adolescents are indeed better at predicting decisions based on naturally occurring human motivations compared to non-human motivations, we also included two players with artificial motivations that do not typically occur in human behavior and that were irrational in the sense of going against the Nash equilibrium. The first of these artificial motivations was inverse greed, which involved cooperating when the greedy player would defect. The second artificial motivation was inverse risk aversion, in which the player defected in situations where a risk-averse player would cooperate. By using the inverse of the human motivations, we ensured that the human and artificial motivations had the same complexity and that cooperation and defection each occurred on 50% of trials.

#### Manipulation check: Self-reported inferred motives

As a manipulation check, participants were asked to describe the player’s strategy after completing the 16 rounds. This was an open-ended question, and participants could type their answers. We used ChatGPT (version *GPT-5.4)* to first translate the answers from Dutch to English (data available on OSF https://osf.io/p5h4j/). Next, we asked GPT to provide a summary per motive type based on the English descriptions. The prompt was as follows:

“*Here is a dataset with descriptions of inferred behavioural economic game strategies, divided into four categories. Please provide a summary of the most likely inferred strategy per category based on the descriptions.*”

Note that we purposefully designed the prompt using “category” instead of giving it the motive name to avoid introducing bias. The motive types and names were not disclosed to the LLM. The output was as follows:

***“Category 1* (greedy). *Most likely inferred strategy***: *payoff maximization focused on the highest possible personal gain. Participants often described this player as going for “the highest amount,” “the most money,” or the “highest possible number,” often without much regard for the opponent. The strategy seems to be seen as greedy / all-or-nothing / risk-seeking, with some comments suggesting the player simply chose the option with the largest potential reward.*

#### Best summary label: Maximize own payoff / highest possible reward

***Category 2* (risk averse). *Most likely inferred strategy***: *self-interested but cautious optimization. This category is described much more often as safe, smart, and aimed at avoiding zero or very low outcomes. Respondents frequently mention that this player tries to get as much as possible while taking the other player’s likely choice into account. So this looks like a strategic, risk-averse self-interest pattern rather than pure greed.*

#### Best summary label: Safe self-interest / strategic maximization with risk avoidance

***Category 3* (inverse risk averse). *Most likely inferred strategy***: *low-payoff or opponent-harming strategy, but perceived as highly unclear or random.*

This category stands out because many respondents say they had no idea, found it strange, unpredictable, or thought there was no strategy. Among the interpretations that do recur, the strongest themes are choosing the lowest option, trying to make the other earn as little as possible, or producing as little profit as possible overall. So the dominant impression is either spiteful minimization or random/unintelligible play.

#### Best summary label: Minimizing outcomes / possibly spiteful, but largely perceived as random

***Category 4* (inverse greed)*. Most likely inferred strategy***: *fair/equality-seeking strategy, though also seen as unpredictable.*

A substantial number of responses mention sharing fairly, keeping the difference small, getting equal outcomes, or maximizing points together. At the same time, many people also say they could not detect a clear pattern and found the choices odd or inconsistent. Still, compared with the other categories, the clearest positive theme is fairness / equality / prosocial balance.

#### Best summary label: Fairness or equality seeking

One important caveat is that **Categories 3 and 4 appear more ambiguous** than 1 and 2. Category 3 is especially dominated by confusion, while Category 4 mixes fairness impressions with unpredictability.”

This result shows that participants’ interpretation of the motives closely matched with our psychological interpretation of greed and risk averse motives used in our manuscript. It also confirms that the inverse motives are harder to interpret for participants despite being statistically equally complex.

#### Questionnaires

We used self-reports to explore whether individual differences in prior (adverse) childhood experiences affect cognitive empathy and their relationship with latent motive inference. Participants filled out the Neighborhood Violence Scale^55^ and the abbreviated Parental Harshness Questionnaire.^56^ These scales measure exposure to violence in the neighborhood as well as exposure to violence caused by one or both parents. We combined the scores of these scales as a measure of the harshness of adolescents' social environments. To explore biases caused by social anxiety symptoms, we used the Social Anxiety Scale for Adolescents,^68^ and the perspective-taking subscale of the Interpersonal Reactivity Index (IRI)^69^ as a measure of cognitive empathy.

#### Computational models

##### Motivation-based model

Our main aim was to investigate what generalizable information adolescents infer to make predictions about others’ behavior in new social contexts. Since this is not directly observable from average task accuracy, we fitted various feature-based reinforcement learning models. Each model formalizes a psychologically plausible hypothesis about the latent motives or other types of latent structure (the ‘features’) that participants may be inferring from the choices of the player to inform their predictions about what the player may choose in a new trial. Evaluating the best-fitting model allows us to examine which latent motives or structures were inferred by each participant individually.

These computational models are described in more detail in van Baar et al.,^5^ but the main ideas are reiterated here for clarity. The main model, called the Motivation-Based Model, reflects learning by inferring the psychological motivations of player 1. The model presupposes a set of latent motives, such as risk aversion and greed, which map potential payoffs onto actions, and assigns weights to these motives through a reinforcement learning process. Importantly, inferring such an underlying motivation supports generalization across novel situations in the game space (i.e., new combinations of S and T) as one motivation results in sharing in one type of game and keeping in another. This model, therefore, allows us to test whether adolescents infer latent motivations and use these to make more accurate predictions.

The Full Motivation-Based Model included four motives: greed, risk aversion, cooperativeness, and expected value maximization (EV). These reflect common motivations in human social decision-making.^16^^,^^18^^,^^70^^,^^71^ Using the same task, van Baar et al.^5^ found that these four motives (greed, risk aversion, cooperativeness, and EV) outperformed 5,000 alternative sets of randomly generated pseudo motives, demonstrating the relevance of the four canonical motives to the social prediction task.

We tested two different versions of the Motivation-Based Model. The full version includes all four motives outlined above. The second version is the Motivation Subset-Based Model, which includes only a subset of these motives. For each subject, the subset is determined by testing each possible combination of the four motives and selecting the combination with the best fit. This approach allows us to detect which and how many motives are considered by each subject, which enables testing for differences between subjects (e.g. by age). For example, younger individuals might only infer one motive while the number of inferred motives might increase with age. This approach thereby reveals how generalization might vary as a function of age during adolescence.

Formally, the agent’s predictions about player 1’s decision to share are given by a value function based on the weighted average of the predictions generated by each included motive. Specifically, the current value of predicting that player 1 will share (*V*_share_) is:

(Equation 1) $$V_{share}=\sum_{i=1}^{k}w_{i\;}M_{i}\left(S,T\right)$$

where *M*_*i*_(*S*,*T*) is the motivation’s prediction at the current *S* and *T* values, this is 1 for share and −1 for keep; *w*_*i*_ is the weight of motivation *i*; *i* is the motivation number; *k* is the total number of motivations. The probability of predicting that player 1 will share (*P*_share_) is then given by a softmax function:

(Equation 2) $$P_{share}=\frac{e^{\beta V_{share}}}{1+e^{\beta V_{share}}}$$

where *β* is the inverse temperature parameter which allows for decision noise. During learning, the agent updates its predictions about player 1’s choice based on a prediction error PE, which is the difference between the agent’s predicted probability of player 1 choosing to share and player 1’s actual choice:

(Equation 3) $$PE=choice-P_{share}$$

where choice = 1 if player 1 shared and 0 if they decided to keep. At each time point, the weight *w*_*i*_ is updated based on *PE* and the learning rate *α* for all motivations *i* from 1 to *k*:

(Equation 4) $$w_{i,t+1}=w_{i,t}+\alpha \times PE\times M_{i}\left(S,T\right)$$

where the updated weight is *w*_i,t+1._ As described earlier, *M*_*i*_(*S*,*T*) = 1 if the prediction for a given motivation would be to share and −1 if it is to keep. By multiplying *M*_*i*_(*S*,*T*) with the prediction error, the motivation’s weight increases if the prediction associated with that motivation was correct, i.e., if *M*_*i*_(*S*,*T*) and PE have the same sign. The weight for a given motivation decreases if the motivation’s prediction was incorrect, i.e., when *M*_*i*_(*S*,*T*) and PE have opposite signs. Time *t* is explicitly mentioned here to distinguish between the current weight *w*_i,t_ and updated weight *w*_i,t+1__._ However, time *t* is left implicit in other parts of Equations 1, 2, 3, and 4.

##### Alternative models

Following van Baar et al.,^5^ we tested two alternative models that are psychologically plausible. The first model, called the Game Type-Based Model, is similar to the main model. Instead of the four motivations, it tests if participants generalize inferences about player 1’s behavior within each game type, such as the Prisoner’s Dilemma (see Figure 1B for an overview of all 4 game types and their mapping onto combinations of S and T). This strategy could be successful given that player 1’s behavior is indeed consistent within each game type (Figure 1C). However, this model does not allow for generalization of a motive between game types like the Motivation-Based Models do. Formally, the Game Type-Based Model is identical to the main model, except that instead of the motivations *M*_*i*_(*S*,*T*) it uses the four game types *G*_*i*_(*S*,*T*). More specifically, the game types were Stag Hunt, Harmony, Snow Drift, and Prisoner's Dilemma.

The second alternative model tests whether adolescents make predictions without generalizing across the specific contextual features of the games. In this model, called the No Structure Model, the agent treats each unique combination of S and T as independent. In addition, one intercept represents the probability of cooperation across the entire game space (i.e. one all-encompassing latent structure) and generalizes across all games equally.

All models were recoverable and the parameters were identifiable as reported in van Baar et al.^5^ All models were fitted for each participant individually using the fmincon function in MATLAB 2023b (MathWorks).

#### Model fitting procedure

The models were fitted for each participant individually using the fmincon function in MATLAB (MathWorks). The Motivation-Based Model had six free parameters: four motive weights, *α* and *β.* The Game Type-Based Model had seven free parameters: four game type weights, one intercept, *α* and *β*, and the No Structure Model had nineteen free parameters: sixteen feature weights corresponding to S and T combinations, one intercept, *α* and *β*. Unlike the rest of the free parameters, the weights were reset to random *a priori* values at the start of each block (when switching to a new player).

Consistent with van Baar et al.,^5^ we fitted the models by minimizing the sum of squared errors, computed by comparing the model prediction *P*_share_ with the prediction *X* which was weighted by the participant’s indicated confidence during the task (scaled between 0 and 1), such that predictions made with less confidence were given less weight in the fitting procedure:

(Equation 5) $$x=\left\{ \begin{array}{c} \frac{1+confidence}{2},cooperate\\ \frac{1-confidence}{2},defect \end{array}\right.$$

(Equation 6) $$SSE=\sum_{t=1}^{n}{\left(x-P_{cooperate}\right)}^{2}$$

Finally, model comparisons were performed based on the Bayesian Information Criterion (BIC) to identify the winning model across participants.

The BIC was derived from the log-likelihood (*LL*) by adding a penalty for model complexity expressed as the number of parameters (k):

(Equation 7) $$LL=\sum_{t=1}^{n}\log\frac{1}{sigma\sqrt{2\pi }}e^{\frac{{-\left(x-P_{cooperate}\right)}^{2}}{{2\sigma }^{2}}}$$

(Equation 8) BIC=−2LL+(k+2)×logn

### Quantification and statistical analysis

#### Primary behavioral analyses

First, we tested if participants' prediction accuracy across the task and per player type was better than chance, using a two-tailed one-sample t-test against 0.5 (as the choice was binary, i.e., predicting sharing or keeping). Because each of the observed players cooperates on half of the trials, prediction accuracy significantly above 50% would suggest that participants are learning elements of a latent structure underlying the players' choices and generalizing that knowledge across games to inform their predictions. Additionally, we tested whether participants hold prior expectations about player behavior to inform their predictions. To do this, we used a within-subjects t-test comparing prediction accuracy in human motive blocks and artificial motive blocks, where higher accuracy in the human motive blocks would suggest that participants hold prior expectations. We expected participants to hold prior expectations about player behavior, learn elements of a latent structure underlying players' choices, and generalize this information across trials to inform their predictions.

Next, we tested whether learning about others (operationalized as task performance) improves with age using a binomial Generalized Linear Mixed Model (GLMM) with the ‘glmer’ function in the R package (lme4). We used trial-wise accuracy (correct vs incorrect prediction) as the outcome variable. We tested the following predictors: age, trial number per player, their interaction, player type (greedy, risk averse, and their inverse motivations), with a random intercept per participant. Our preregistered hypothesis was that learning about others improves with age throughout adolescence.

We then tested how prediction accuracy varied when predicting the choices of each of the four player profiles across the four types of games using a repeated-measures ANOVA, followed by Bonferroni corrected pairwise comparisons. Both tests used accuracy as the outcome variable and player type and game type as predictors.

#### Computational model comparison

To compare the fit of each model at the participant level, we used a pairwise BIC comparison. For each model pair, we computed the BIC difference per participant (ΔBIC) and determined which of the models tested fitted the data best (i.e., had the lowest BIC). We then calculated the mean ΔBIC across participants and counted the wins and losses for each model pair across participants. Finally, we assess the reliability of the mean ΔBIC using a percentile bootstrapped 95% CI (10,000 resamples), where CIs not overlapping zero indicate a reliable difference in model fit.

To further validate the model comparison, we carried out a random-effects Bayesian Model Selection (RFX-BMS) analysis using the VBA MATLAB package.^32^ To account for differences in model complexity, log model evidence was approximated using the BIC (log evidence ≈ −BIC/2). RFX-BMS estimates the posterior probability of each model at the individual level and then uses these estimates to infer the frequency with which each model best describes behavior at the group level. We summarize this information through three statistics: (1) the expected model frequency (r), which reflects the estimated proportion of the population best described by a given model; (2) the protected exceedance probability (PEP), which reflects the corrected probability that a given model is more frequent than all others in the population; (3) the Bayesian omnibus risk (BOR), which reflects the probability that all models are equally frequent. A high r and PEP together with a low BOR would indicate support for a given model.

#### Additional analyses

##### Age differences in inferred motivations

To test age differences in the number of inferred motivations as well as the kinds of inferred motivations, we used a partial proportional odds model, using the vglm function of the VGAM package in R, to test if the number of inferred motivations varied as a function of age. Similarly, a series of logistic regression analyses was used to test the role of age in the probability of expecting a specific motive to drive players' behavior, with a separate logistic regression analysis used for each of the four canonical motives included in the computational modelling procedure. We tested variations of each regression model with linear, quadratic, and cubic age effects and compared them using a likelihood ratio test (LRT) to determine the most appropriate age effect for each motive (LRT results can be found in supplement 2).

##### Self-anchoring analyses

During the task explanation, participants played one example of each game type from their own perspective, indicating whether they would keep or share monetary units. We used these responses to determine participants' own motive when playing the economic games themselves. To do this, we compared participants' responses across the four game types with the response patterns expected for each motive.

Then, we tested whether participants' own motives changed as a function of age using a multinomial logistic regression model comparison analysis. To test for self-anchoring, we examined whether participants' accuracy was higher when they predicted the choices of a player whose behavior matched their own motivation. To do this, we used a linear regression analysis in which participants' own motive and age were used as predictors of accuracy. We conducted separate regression analyses for predictions about the risk-averse and greedy players.

Additionally, we explored the overlap between the motives participants' considered (cooperativeness, greed, risk aversion, expected value) in their mental model according to the winning computational model and their own preferred motives. To do this, we computed the percentage of participants who preferred the four motives tested in the computational model and considered them according to the winning model (Figure S3).

##### Individual differences analyses

To further investigate the factors that may affect adolescents' social inference skills, we explored the relationship between mental model complexity, preferring the risk averse motive, environmental harshness, social anxiety trait, and cognitive empathy (see section 2.3.2 for measurement description). To this end, we used multiple binary logistic regressions controlling for age.

## References

[1] Crone E.A., Dahl R.E. (2012). Understanding adolescence as a period of social–affective engagement and goal flexibility. Nat. Rev. Neurosci..

[2] Nelson E.E., Jarcho J.M., Guyer A.E. (2016). Social re-orientation and brain development: An expanded and updated view. Dev. Cogn. Neurosci..

[3] Van Den Bos W. (2013). Neural Mechanisms of Social Reorientation across Adolescence. J. Neurosci..

[4] Ma I., Westhoff B., Van Duijvenvoorde A.C.K. (2022). Uncertainty about others’ trustworthiness increases during adolescence and guides social information sampling. Sci. Rep..

[5] Van Baar J.M., Nassar M.R., Deng W., FeldmanHall O. (2022). Latent motives guide structure learning during adaptive social choice. Nat. Hum. Behav..

[6] Dahl R.E., Allen N.B., Wilbrecht L., Suleiman A.B. (2018). Importance of investing in adolescence from a developmental science perspective. Nature.

[7] Krabbendam L., Sijtsma H., Crone E.A., Van Buuren M. (2024). Trust in adolescence: Development, mechanisms and future directions. Dev. Cogn. Neurosci..

[8] Ma I., Sanfey A.G., Ma W.J. (2020). The social cost of gathering information for trust decisions. Sci. Rep..

[9] Fett A.K.J., Shergill S.S., Gromann P.M., Dumontheil I., Blakemore S.J., Yakub F., Krabbendam L. (2014). Trust and social reciprocity in adolescence – A matter of perspective-taking. J. Adolesc..

[10] Schreuders E., Van Buuren M., Walsh R.J., Sijtsma H., Hollarek M., Lee N.C., Krabbendam L. (2024). Learning whom not to trust across early and middle adolescence: A longitudinal neuroimaging study to trusting behavior involving an uncooperative other. Child Dev..

[11] Sijtsma H., Lee N.C., Braams B.R., Hollarek M., Walsh R.J., Van Buuren M., Krabbendam L. (2023). The development of adolescent trust behavior. J. Exp. Child Psychol..

[12] Lee N.C., Jolles J., Krabbendam L. (2016). Social information influences trust behaviour in adolescents. J. Adolesc..

[13] Balliet D., Lindström B. (2023). Inferences about interdependence shape cooperation. Trends Cogn. Sci..

[14] Frolichs K.M.M., Rosenblau G., Korn C.W. (2022). Incorporating social knowledge structures into computational models. Nat. Commun..

[15] Peysakhovich A., Nowak M.A., Rand D.G. (2014). Humans display a ‘cooperative phenotype’ that is domain general and temporally stable. Nat. Commun..

[16] Poncela-Casasnovas J., Gutiérrez-Roig M., Gracia-Lázaro C., Vicens J., Gómez-Gardeñes J., Perelló J., Moreno Y., Duch J., Sánchez A. (2016). Humans display a reduced set of consistent behavioral phenotypes in dyadic games. Sci. Adv..

[17] Camerer C.F. (2011).

[18] Fehr E., Fischbacher U. (2003). The nature of human altruism. Nature.

[19] Engel C., Zhurakhovska L. (2016). When is the risk of cooperation worth taking? The prisoner’s dilemma as a game of multiple motives. Appl. Econ. Lett..

[20] Capraro V., Jordan J.J., Rand D.G. (2014). Heuristics guide the implementation of social preferences in one-shot Prisoner’s Dilemma experiments. Sci. Rep..

[21] Fett A.K.J., Gromann P.M., Giampietro V., Shergill S.S., Krabbendam L. (2014). Default distrust? An fMRI investigation of the neural development of trust and cooperation. Soc. Cogn. Affect. Neurosci..

[22] Dumontheil I., Apperly I.A., Blakemore S.J. (2010). Online usage of theory of mind continues to develop in late adolescence. Dev. Sci..

[23] Guazzelli Williamson V., Barendse M.E.A., Chavez S.J., Flournoy J.C., Cheng T.W., Cosme D., Byrne M.L., Allen N.B., Pfeifer J.H. (2025). A longitudinal neuroimaging study of adolescent girls’ mentalizing and perspective-taking tendencies. Dev. Cogn. Neurosci..

[24] Van Der Graaff J., Carlo G., Crocetti E., Koot H.M., Branje S. (2018). Prosocial Behavior in Adolescence: Gender Differences in Development and Links with Empathy. J. Youth Adolesc..

[25] Van Lissa C.J., Hawk S.T., De Wied M., Koot H.M., Van Lier P., Meeus W. (2014). The longitudinal interplay of affective and cognitive empathy within and between adolescents and mothers. Dev. Psychol..

[26] De Lillo M., Ferguson H.J. (2023). Perspective-taking and social inferences in adolescents, young adults, and older adults. J. Exp. Psychol. Gen..

[27] Hall H.K., Millear P.M.R., Summers M.J., Isbel B. (2021). Longitudinal Research on Perspective Taking in Adolescence: A Systematic review. Adolesc. Res. Rev..

[28] Gutiérrez-García A., Calvo M.G. (2017). Social anxiety and threat-related interpretation of dynamic facial expressions: Sensitivity and response bias. Pers. Individ. Dif..

[29] Kim S.Y., Shin J.E., Lee Y.I., Kim H., Jo H.J., Choi S.H. (2018). Neural evidence for persistent attentional bias to threats in patients with social anxiety disorder. Soc. Cogn. Affect. Neurosci..

[30] Miller L.E. (2015). Perceived threat in childhood. Trauma Violence Abuse.

[31] Pineles S.L., Mineka S. (2005). Attentional biases to internal and external sources of potential threat in social anxiety. J. Abnorm. Psychol..

[32] Daunizeau J., Adam V., Rigoux L. (2014). VBA: A probabilistic treatment of nonlinear models for neurobiological and behavioural data. PLoS Comput. Biol..

[33] Blakemore S.J., Mills K.L. (2014). Is adolescence a sensitive period for sociocultural processing?. Annu. Rev. Psychol..

[34] Hofmans L., Van Den Bos W. (2022). Social learning across adolescence: A Bayesian neurocognitive perspective. Dev. Cogn. Neurosci..

[35] Topel S., Ma I., Sleutels J., Van Steenbergen H., De Bruijn E.R.A., Van Duijvenvoorde A.C.K. (2023). Expecting the unexpected: a review of learning under uncertainty across development. Cogn. Affect. Behav. Neurosci..

[36] Towner E., Chierchia G., Blakemore S.J. (2023). Sensitivity and specificity in affective and social learning in adolescence. Trends Cogn. Sci..

[37] Wilbrecht L., Davidow J.Y. (2024). Goal-directed learning in adolescence: neurocognitive development and contextual influences. Nat. Rev. Neurosci..

[38] Van Baar J.M., Klaassen F.H., Ricci F., Chang L.J., Sanfey A.G. (2020). Stable distribution of reciprocity motives in a population. Sci. Rep..

[39] Gweon H., Asaba M., Bennett-Pierre G., Gweon H., Asaba M., Bennett-Pierre G. (2017). Proceedings of the 39th Annual Meeting of the Cognitive Science Society.

[40] Jara-Ettinger J., Gweon H., Tenenbaum J.B., Schulz L.E. (2015). Children’s understanding of the costs and rewards underlying rational action. Cognition.

[41] Liu W., Shah N., Ma I., Rosenblau G. (2024). Strategic social decision making undergoes significant changes in typically developing and autistic early adolescents. Dev. Sci..

[42] Kilford E.J., Garrett E., Blakemore S.J. (2016). The development of social cognition in adolescence: An integrated perspective. Neurosci. Biobehav. Rev..

[43] Rosenblau G., Korn C.W., Dutton A., Lee D., Pelphrey K.A. (2021). Neurocognitive mechanisms of social inferences in typical and autistic adolescents. Biol. Psychiatry Cogn. Neurosci. Neuroimaging.

[44] Choudhury S., Blakemore S.J., Charman T. (2006). Social cognitive development during adolescence. Soc. Cogn. Affect. Neurosci..

[45] Valle A., Massaro D., Castelli I., Marchetti A. (2015). Theory of Mind Development in Adolescence and Early Adulthood: The growing complexity of Recursive Thinking ability. Eur. J. Psychol..

[46] Rosenblau G., Korn C.W., Pelphrey K.A. (2018). A computational account of optimizing social predictions reveals that adolescents are conservative learners in social contexts. J. Neurosci..

[47] Baker C.L., Saxe R., Tenenbaum J.B. (2009). Action understanding as inverse planning. Cognition.

[48] Liu S., Ullman T.D., Tenenbaum J.B., Spelke E.S. (2017). Ten-month-old infants infer the value of goals from the costs of actions. Science.

[49] Gweon H., Asaba M., Bennett-Pierre G. (2017). Proceedings of the Annual Meeting of the Cognitive Science Society, 39.

[50] Ondobaka S., De Lange F.P., Newman-Norlund R.D., Wiemers M., Bekkering H. (2012). Interplay between action and movement intentions during social interaction. Psychol. Sci..

[51] Sebanz N., Knoblich G., Prinz W. (2003). Representing others’ actions: just like one’s own?. Cognition.

[52] Vesper C., Abramova E., Bütepage J., Ciardo F., Crossey B., Effenberg A., Hristova D., Karlinsky A., McEllin L., Nijssen S.R.R. (2016). Joint Action: Mental Representations, Shared Information and General Mechanisms for Coordinating with Others. Front. Psychol..

[53] Tamir D.I., Mitchell J.P. (2013). Anchoring and adjustment during social inferences. J. Exp. Psychol. Gen..

[54] Frith C.D., Frith U. (2012). Mechanisms of social cognition. Annu. Rev. Psychol..

[55] Frankenhuis W.E., Roelofs M.F.A., De Vries S.A. (2018). Does exposure to psychosocial adversity enhance deception detection ability?. Evol. Behav. Sci..

[56] Ellis B.J., Schlomer G.L., Tilley E.H., Butler E.A. (2012). Impact of fathers on risky sexual behavior in daughters: A genetically and environmentally controlled sibling study. Dev. Psychopathol..

[57] Bandura A. (1977). Self-efficacy: Toward a unifying theory of behavioral change. Psychol. Rev..

[58] Pinho A.d.S., Molleman L., Braams B.R., Van Den Bos W. (2021). Majority and popularity effects on norm formation in adolescence. Sci. Rep..

[59] Thornton A., Clutton-Brock T. (2011). Social learning and the development of individual and group behaviour in mammal societies. Philos. Trans. R. Soc. Lond. B Biol. Sci..

[60] Nussenbaum K., Scheuplein M., Phaneuf C.V., Evans M.D., Hartley C.A. (2020). Moving Developmental research online: Comparing In-Lab and Web-Based studies of Model-Based reinforcement learning. Collabra Psychology.

[61] Cruz S., Sousa M., Marchante M., Coelho V.A. (2023). Trajectories of social withdrawal and social anxiety and their relationship with self-esteem before, during, and after the school lockdowns. Sci. Rep..

[62] Houghton S., Kyron M., Hunter S.C., Lawrence D., Hattie J., Carroll A., Zadow C. (2022). Adolescents’ longitudinal trajectories of mental health and loneliness: The impact of COVID-19 school closures. J. Adolesc..

[63] Kozák M., Bakken A., Von Soest T. (2023). Psychosocial well-being before, during and after the COVID-19 pandemic: a nationwide study of more than half a million Norwegian adolescents. Nat. Ment. Health.

[64] Kuper-Smith B.J., Korn C.W. (2025). Loss avoidance during social interactions. Commun. Psychol..

[65] Wang X., Navarro-Martinez D. (2023). Bridging the gap between the economics lab and the field: Dictator games and donations. Judgm. Decis. Mak..

[66] Liu W., Mark I., Korn C.W., Rosenblau G. (2025). Social Context Matters—Characterizing adolescent cooperation strategies when perceiving the other as a peer versus computer partner. J. Adolesc..

[67] Petersen A.C., Crockett L., Richards M., Boxer A. (1988). A self-report measure of pubertal status: Reliability, validity, and initial norms. J. Youth Adolesc..

[68] La Greca A.M., Lopez N. (1998). Social Anxiety Among Adolescents: Linkages with Peer Relations and Friendships. J. Abnorm. Child Psychol..

[69] Davis M.H. (1983). Measuring individual differences in empathy: Evidence for a multidimensional approach. J. Pers. Soc. Psychol..

[70] Bernoulli D. (1954). Exposition of a new theory on the measurement of risk. Econometrica.

[71] Von Neumann J., Morgenstern O. (1944).

