# Supplementary material for: Adolescents infer latent motives to build generalizable models of others’ behavior
Source: iScience. 2026 Oct 27;29(11):118039. doi: 10.1016/j.isci.2026.118039 (PMC13735002; doi:10.1016/j.isci.2026.118039)
Supplement: Supplementary file 1 [file mmc1.pdf]

iScience, Volume *29*

Supplemental information

Adolescents infer latent motives to build generalizable models of others' behavior

Jaime Vigil-Escalera, Jeroen van Baar, Lucas Molleman, Anna C.K. van Duijvenvoorde, and Ili Ma

SUPPLEMENTARY MATERIALS

1. Game type accuracy

Supplementary Figure 1

Mean Accuracy by Player Type and Game Type

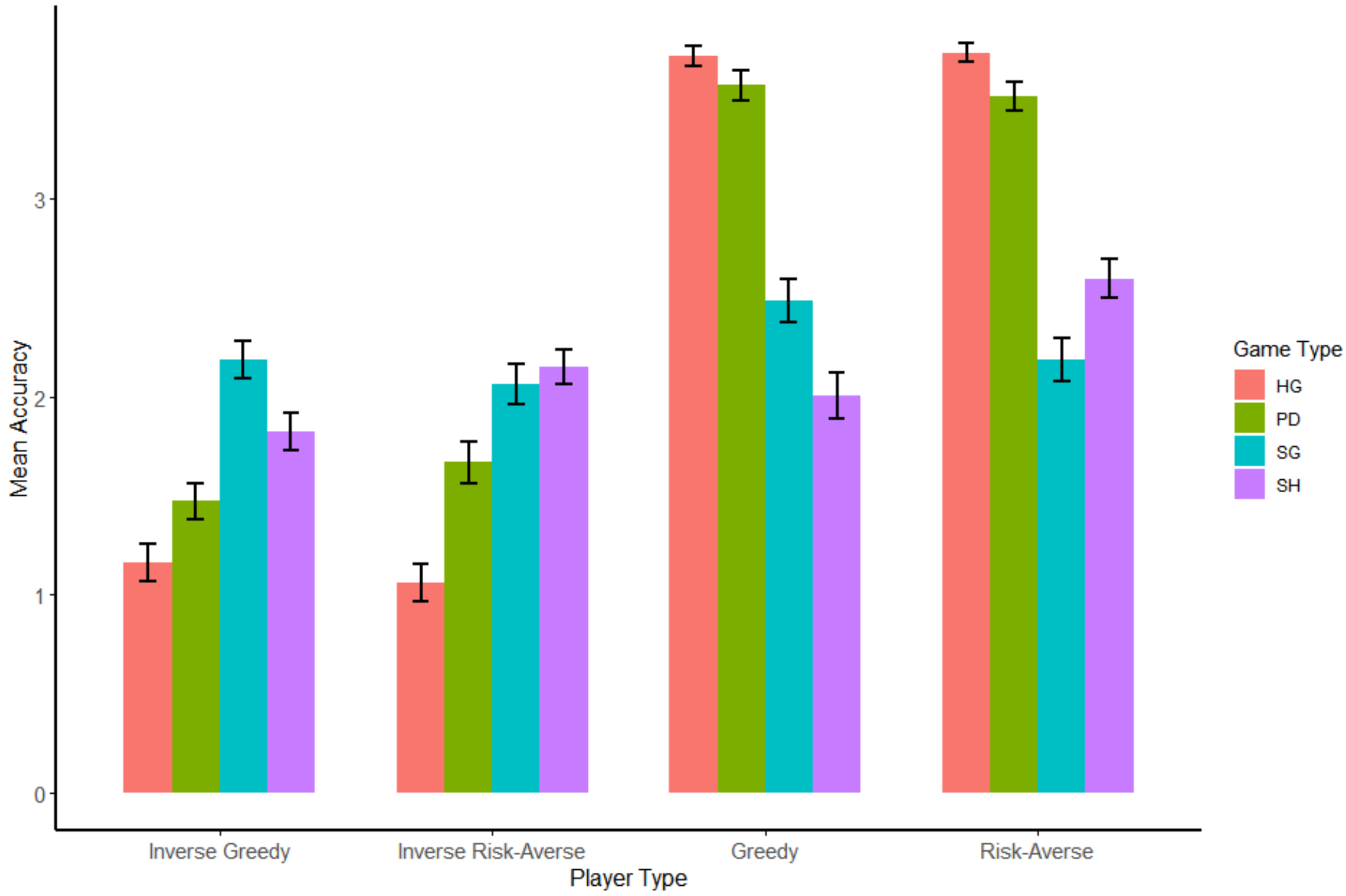

Note: HG stands for harmony game, PD for prisoners' dilemma, SG for snowdrift game, and SH for stag hunt game. Error bars indicate the standard deviation.

2. Self-anchoring

Supplementary Table 1

Changes in own motive as a function of age

	β	Std.Error	p value
Coop_(Intercept)	12.294	0.015	<0.001
Greed_(Intercept)	-30.501	0.018	<0.001
Nash_(Intercept)	-3.953	0.015	<0.001
Risk_(Intercept)	-26.028	0.016	<0.001
Coop_age	-1.957	0.108	<0.001
Greed_age	3.731	0.143	<0.001
Nash_age	0.504	0.106	<0.001
Risk_age	3.057	0.128	<0.001
Coop_I(age^2)	0.065	0.006	<0.001
Greed_I(age^2)	-0.117	0.009	<0.001
Nash_I(age^2)	-0.021	0.007	<0.01
Risk_I(age^2)	-0.092	0.008	<0.001
Coop_(Intercept)	12.294	0.015	<0.001
Greed_(Intercept)	-30.501	0.018	<0.001
Nash_(Intercept)	-3.953	0.015	<0.001
Risk_(Intercept)	-26.028	0.016	<0.001
Coop_age	-1.957	0.108	<0.001
Greed_age	3.731	0.143	<0.001

Supplementary Figure 2.

Predicted Probabilities of Self-Type by Age

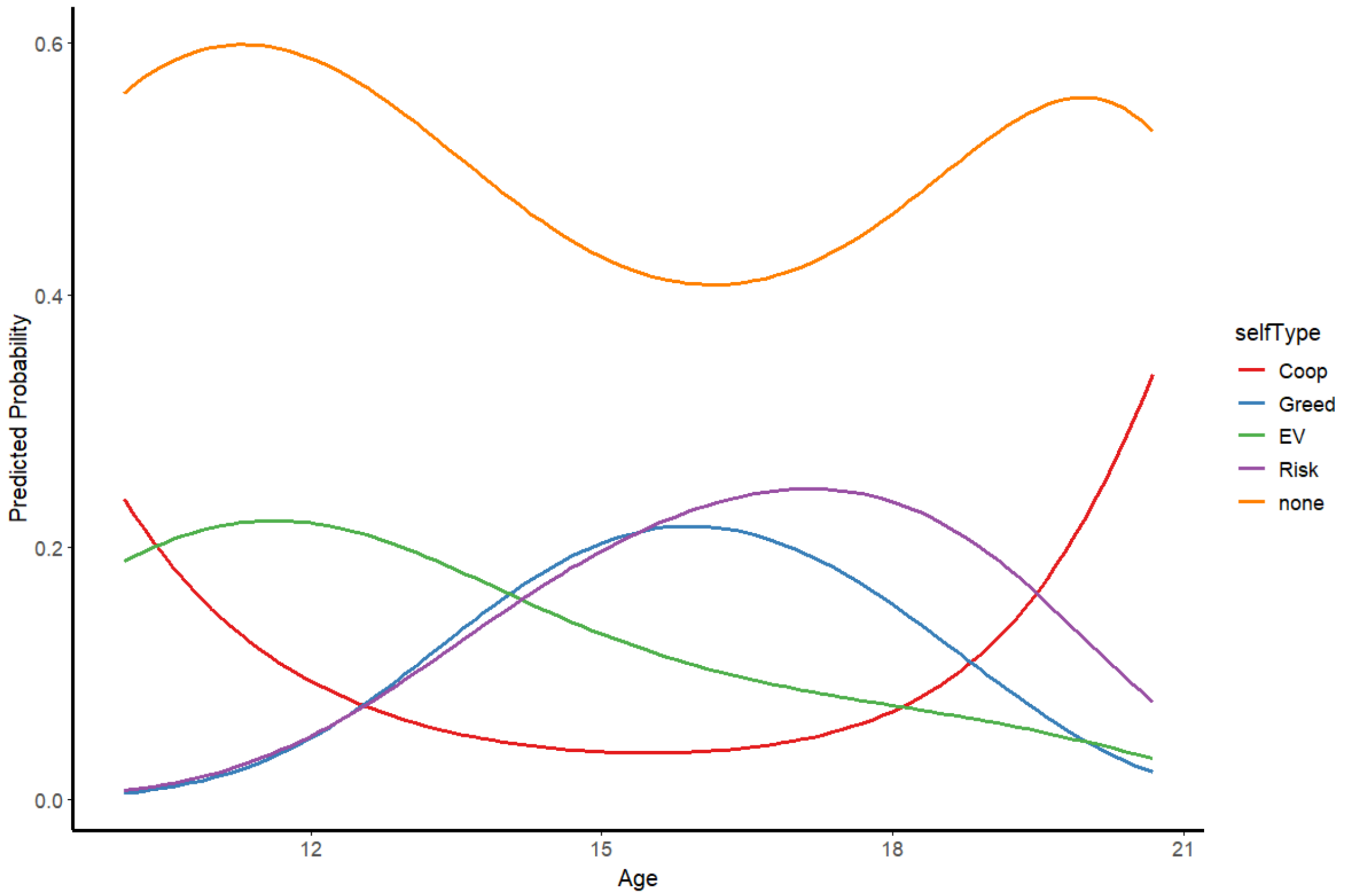

Note: The lines represent age-related changes in the probability that participants themselves adhered to a strategy across adolescence. The strategies are abbreviated as follows: Coop refers to cooperativeness, Greed refers to the greedy strategy, EV refers to expected value maximization, Risk refers to risk aversion, and none indicates that the participant's strategy deviated from one of the canonical strategies during one or more trials.

Supplementary Figure 3

Overlap between own motive and expected motive

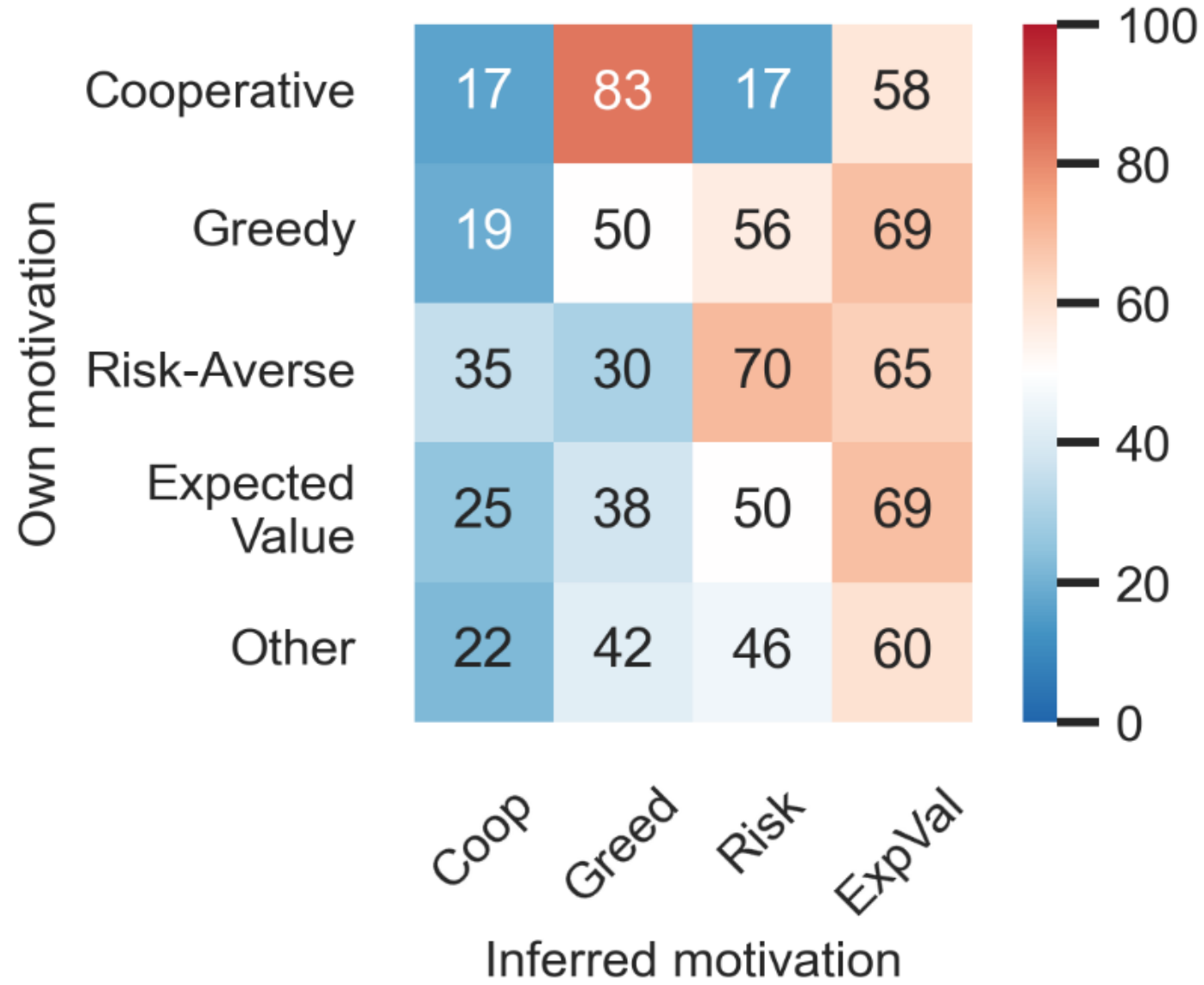

Note: The heatmap shows the overlap, represented with percentages, between inferred motivations according to the computational modelling results and participants' own preferred motivation. Summing up the percentages across rows and columns exceeds 100, because multiple participants inferred more than one motivation. The own motivation labelled "Other" indicates the participants who did not fit into any of the motives tested.

Individual differences

Supplementary Table 2. Multiple binary logistic regression controlling for age

Comparison	term	OR	CI Low	CI High	p value	p value holm
Complexity >1	Harshness	0.975	0.917	1.036	0.409	1.000
Complexity >1	Age	1.166	1.019	1.334	0.025	0.279
Complexity >1	SAS A	0.977	0.952	1.003	0.081	0.566
Complexity >1	Age	1.181	1.030	1.353	0.017	0.204
Complexity >1	Empathy (IRI)	1.025	0.945	1.111	0.558	1.000
Complexity >1	Age	1.143	0.995	1.312	0.058	0.524
Complexity >1	Harshness	0.981	0.907	1.063	0.644	1.000
Self type = risk averse	Age	1.152	0.961	1.381	0.126	0.756
Self type = risk averse	SAS A	0.963	0.929	0.999	0.042	0.421
Self type = risk averse	Age	1.190	0.985	1.436	0.071	0.566
Self type = risk averse	Empathy (IRI)	1.076	0.957	1.211	0.221	1.000
Self type = risk averse	Age	1.103	0.920	1.323	0.289	1.000

Note: Harshness refers to environmental harshness, SAS A refers to the social anxiety scale, Empathy (IRI) refers to the cognitive empathy scale.
